# PIM1 phosphorylates ABI2 to enhance actin dynamics and promote tumor invasion

**DOI:** 10.1083/jcb.202208136

**Published:** 2023-04-12

**Authors:** Corbin C. Jensen, Amber N. Clements, Hope Liou, Lauren E. Ball, Jennifer R. Bethard, Paul R. Langlais, Rachel K. Toth, Shailender S. Chauhan, Andrea L. Casillas, Sohail R. Daulat, Andrew S. Kraft, Anne E. Cress, Cindy K. Miranti, Ghassan Mouneimne, Greg C. Rogers, Noel A. Warfel

**Affiliations:** 1 Cancer Biology Graduate Program, University of Arizona, Tucson, AZ, USA; 2 Department of Cell and Molecular Pharmacology and Experimental Therapeutics, Medical University of South Carolina, Charleston, SC, USA; 3 Department of Medicine, University of Arizona, Tucson, AZ, USA; 4 University of Arizona Cancer Center, Tucson, AZ, USA; 5 Department of Cellular and Molecular Medicine, University of Arizona, Tucson, AZ, USA

## Abstract

Distinguishing key factors that drive the switch from indolent to invasive disease will make a significant impact on guiding the treatment of prostate cancer (PCa) patients. Here, we identify a novel signaling pathway linking hypoxia and PIM1 kinase to the actin cytoskeleton and cell motility. An unbiased proteomic screen identified Abl-interactor 2 (ABI2), an integral member of the wave regulatory complex (WRC), as a PIM1 substrate. Phosphorylation of ABI2 at Ser183 by PIM1 increased ABI2 protein levels and enhanced WRC formation, resulting in increased protrusive activity and cell motility. Cell protrusion induced by hypoxia and/or PIM1 was dependent on ABI2. In vivo smooth muscle invasion assays showed that overexpression of PIM1 significantly increased the depth of tumor cell invasion, and treatment with PIM inhibitors significantly reduced intramuscular PCa invasion. This research uncovers a HIF-1-independent signaling axis that is critical for hypoxia-induced invasion and establishes a novel role for PIM1 as a key regulator of the actin cytoskeleton.

## Teaser

PIM1 kinase promotes prostate cancer invasion by directly phosphorylating ABI2, which increases WAVE regulatory complex activity and enhances actin dynamics to drive tumor cell protrusion. These findings represent the first signal transduction pathway linking hypoxia to the regulation of actin cytoskeletal dynamics and reveal PIM1 as a new target to opposing hypoxia-induced metastasis.

## Introduction

The ability of cancer cells to migrate and invade surrounding tissue is an early and essential step for metastasis, a lethal progression of solid tumors, that accounts for over 90% of cancer-related deaths. This is particularly true for prostate cancer (PCa), where patients have a good prognosis if the tumor remains confined to the primary organ. However, when tumor cells escape the gland, there is a dramatic decrease in the 5-yr survival (from nearly 100 to 30%; Zhang et al., 2021). Although the metastatic process is complex, it is entirely dependent on the ability of cells to migrate. Therefore, identifying new, druggable targets to inhibit cancer cell migration represents a promising strategy to block disease progression and improve prognosis in PCa patients.

Hypoxia, or low oxygen concentration, is a hallmark of solid tumors that is strongly associated with invasion and metastasis (Jensen and Warfel, 2021). In PCa, increased expression of hypoxia-induced proteins correlates with treatment failure, independent of tumor stage, Gleason score, and prostate-specific antigen (PSA) levels (Deep and Panigrahi, 2015). Higher expression of hypoxia-induced genes, such as hypoxia-inducible factor (HIF)-1, vascular endothelial growth factor, and osteopontin, is associated with early biochemical treatment failure and disease recurrence in patients with clinically localized PCa (Vergis et al., 2008). Moreover, hypoxia is an independent predictor of biochemical recurrence and is a better predictor of local recurrence of PCa than PSA levels or Gleason score (Milosevic et al., 2012). Recent work has established a Proviral Integration site for Moloney murine leukemia virus (PIM) 1 kinase as a hypoxia-inducible prosurvival protein (Casillas et al., 2018). PIM1 is a member of a family of oncogenic Ser/Thr kinases whose levels are elevated in many types of solid tumors, particularly PCa. PIM1 levels are elevated in high-grade prostatic intraepithelial neoplasia relative to normal tissue and further elevated in castration-resistant prostate cancer (Dhanasekaran et al., 2001), and high PIM1 expression is associated with resistance to therapy (Logothetis et al., 2018). Notably, PIM1 levels are upregulated in hypoxia in an HIF-independent manner due to increased protein stability (Toth et al., 2022). Functionally, PIM1 has been shown to promote therapeutic resistance and enhance proliferation, survival, and angiogenesis (Toth et al., 2019; Toth and Warfel, 2021). However, a role for PIM1 in tumor cell invasion and metastasis has not been described.

We previously reported that treatment with small-molecule PIM inhibitors significantly reduces metastasis in orthotopic models of prostate and colon cancer (Casillas et al., 2018), but the mechanisms responsible have not been established. Here, we identify PIM1 as a potent driver of hypoxia-induced migration and invasion, key initial steps in the metastatic process. An unbiased proteomic screen performed in hypoxia identified Abl-interactor 2 (ABI2) as a novel substrate for PIM kinases. ABI2 plays an essential role in regulating actin dynamics and cell motility as an integral member of the WAVE regulatory complex (WRC; Dai and Pendergast, 1995). The WRC is a stable heteropentamer of isoforms of the following five proteins: WASp-family verprolin homologous protein (WAVE), Abl-interacting protein (ABI), NCK-associated proteins (NAP), specifically Rac-associated 1 (SRA1), and hematopoietic stem progenitor cell 300 (HSPC300; Rottner et al., 2021). The WRC is held in an inactive conformation until the cell receives signals that initiate Rac binding or phosphorylation by protein kinases, which cause a conformation change in WAVE2 that releases the verprolin, cofilin, and acidic (VCA) domain (Mendoza, 2013). This results in the recruitment of the actin-related protein (ARP) 2/3 complex, which stimulates actin branching and cellular protrusions (Echarri et al., 2004). Our findings reveal that phosphorylation by PIM1 increases ABI2 protein stability, which leads to stabilization of the WRC and enhances tumor cell protrusive activity and invasion. This work provides the first direct link between hypoxia, PIM kinases, and the regulation of the actin cytoskeleton. The identification of this signaling axis poises PIM1 as a critical driver of cancer cell invasion and metastasis and provides a novel target to oppose the prometastatic effect of hypoxia in PCa.

## Results

### PIM1 is critical for hypoxia-induced invasion and cellular protrusions

**Figure S1.**
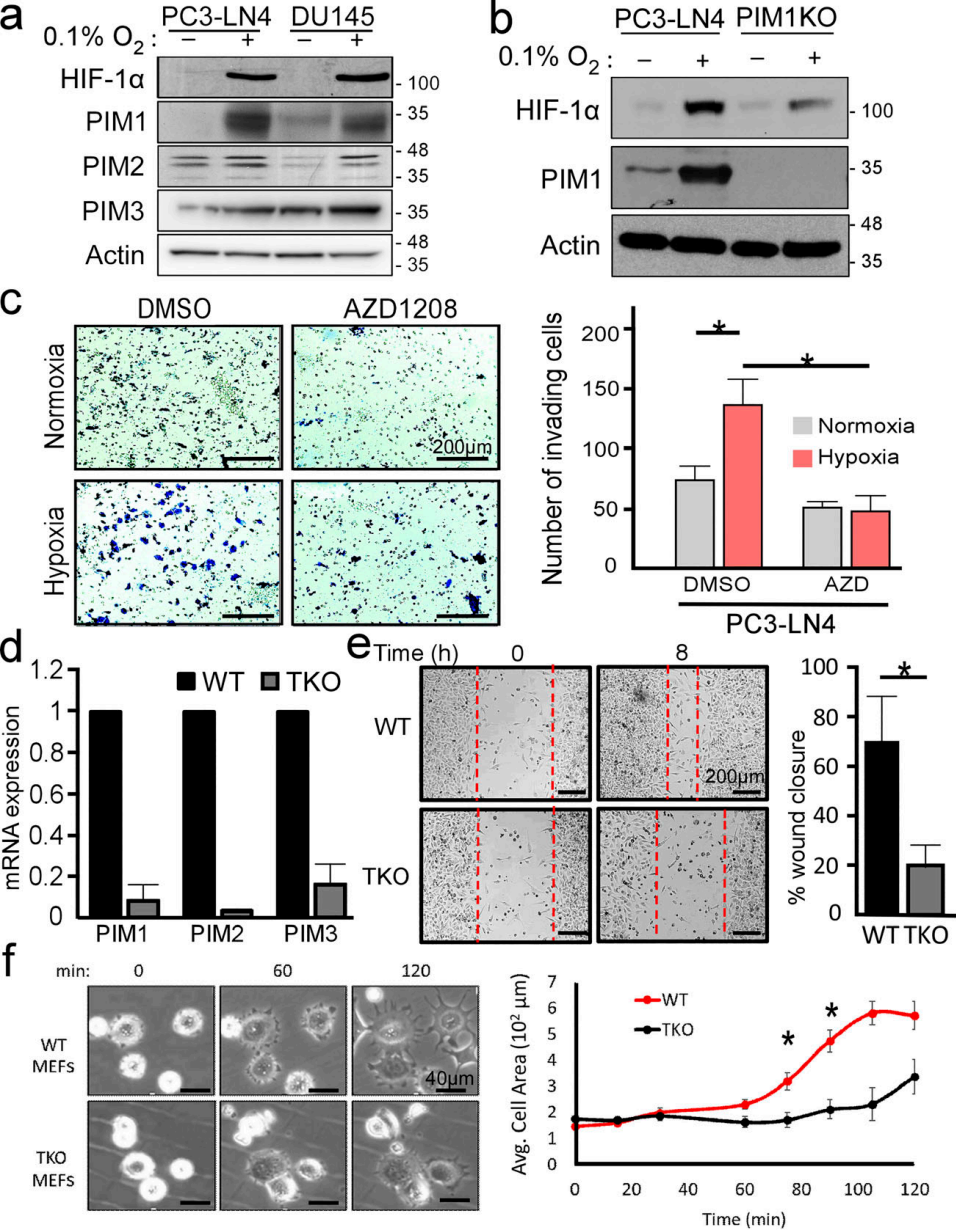
**Loss of PIM inhibits cell motility and spreading. (a)** PIM1/2/3 expression in LN4 and DU145 in normoxia and hypoxia. **(b)** PIM1 expression in LN4 and CRISPR normoxia vs. hypoxia. **(c)** Boyden Chamber invasion assay of PC3-LN4 cells with or without PIM inhibition under normoxic and hypoxic conditions. The number of invading cells was quantified. Error displayed as SEM (*n* = 3; *P < 0.05). Scale bar = 200 µm. **(d)** mRNA expression for PIM1/2/3 in WT and TKO cell lines. **(e)** Wound healing assay and subsequent quantification of percent wound closure in MEFs with intact or complete loss of PIM kinase. Error is SEM (*n* = 3; *P < 0.05). Scale bar = 200 µm. **(f)** WT and TKO cells were placed in dishes and allowed to seed. Cells were imaged every 20 min and average cell area was plotted over time. Error is SEM (*n* = 3; *P < 0.05). Scale bar = 40 µm.

**Figure 1.**
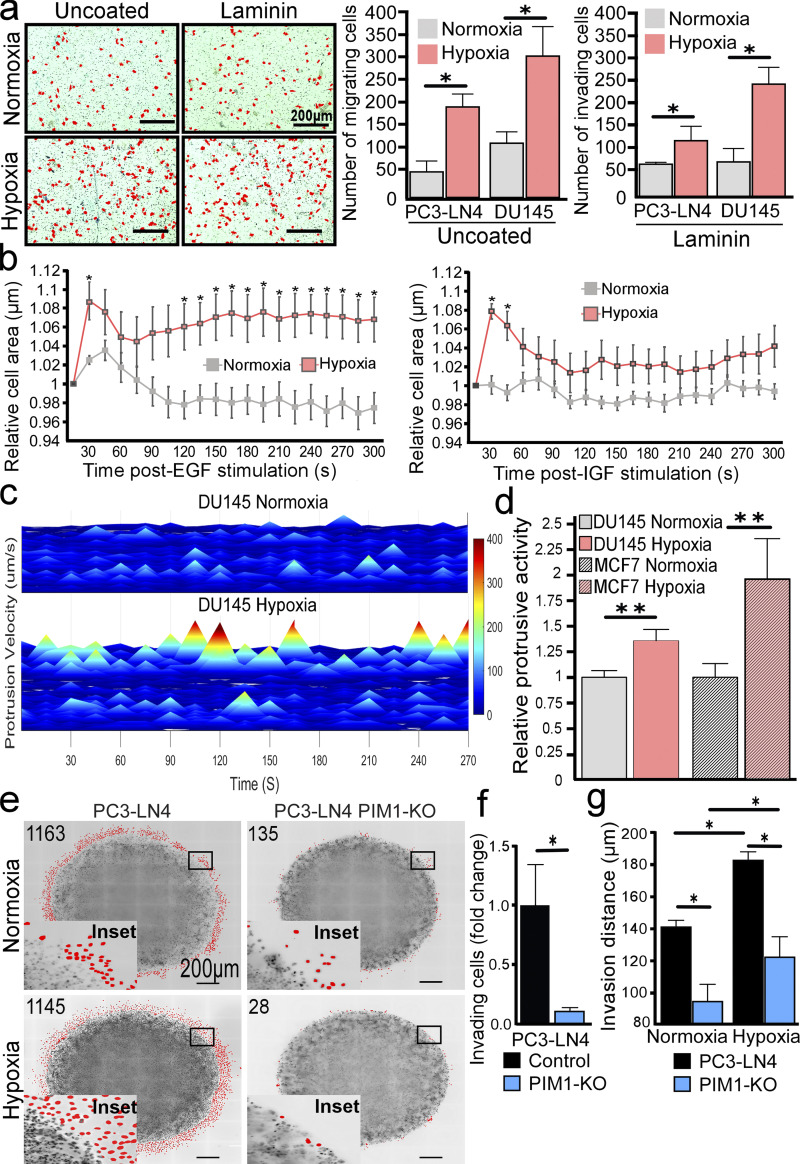
**Hypoxia increases tumor cell invasion and protrusive activity. (a)** Representative images of Boyden chambers invasion assay in normoxia (Nx) or hypoxia (Hx; 1% O_2_), with accompanying quantification of cell number (*n* = 3). **(b)** Quantification of DU145 cell area following stimulation with EGF (5 nM) and IGF (5 nM; *n* > 10 cells/condition). Values are relative to cell area prior to stimulation. **(c)** Representative heatmap of basal (unstimulated) protrusive velocity of DU145 cells in normoxia (*n* = 1,637, *N* = 5) and hypoxia (*n* = 1,286, *N* = 12). **(d)** Quantification of relative protrusion activity in normoxia and hypoxia for DU145 (*n* = 14) and MCF7 (*n* > 9) cells. Error is shown as 95% CI by two-sided Student’s *t* test (*P < 0.05, **P < 0.01). **(e)** Representative images of 3D invasion assays using PC3-LN4 and PC3-LN4 PIM1-KO cells in normoxia and hypoxia. Invading cells are marked in red, and the number of invading cells is indicated in the upper left corner. Scale bar = 200 µm for all images. **(f and g)** Quantification of the total number of invading cells and (g) average invasion distance from the core of PC3-LN4 (*n* = 2,864 Nx and *n* = 2,079 Hx) and PC3-LN4 PIM1-KO (*n* = 255 Nx and *n* = 143 Hx) cells. *N* = 3 biological replicates for all conditions. Error is shown as 95% CI by two-sided Student’s *t* test (*P < 0.05).

Hypoxia is known to promote an aggressive phenotype in solid tumors, as demonstrated by clinical association with heightened metastasis (Rankin and Giaccia, 2016). Although many hypoxia-induced factors can influence metastasis, the effect of hypoxia on early events, such as protrusive activity, has not been well studied. Therefore, we first established the effect of hypoxia on the invasive potential of PCa cell lines. Boyden chamber Transwell assays were performed using PC3-LN4 and DU145 PCa cells. We observed increased levels of PIM1, PIM2, and PIM3 in both cell lines after 4 h hypoxia compared with normoxia (Fig. S1 a). Exposure to hypoxia significantly increased the migration (uncoated) and invasion (laminin-coated) of both cell lines compared with cells maintained in normoxia (Fig. 1 a). Next, we examined whether hypoxia increased cellular protrusion. To this end, we stably transfected DU145 cells with a GFP-tagged Lifeact to label actin. These cells were starved using DMEM with 0.5% FBS for 4 h and then imaged every 15 s over the course of the experiment. To establish a baseline level of protrusive activity, cells were imaged for 5 min prior to stimulation with either epithelial growth factor (EGF; 5 nM) or insulin growth factor (IGF; 5 nM). The protrusion was measured as the change in cell area over time relative to the established baseline. We found that cells conditioned in hypoxia displayed significantly larger increases in cell area in response to EGF or IGF stimulation than cells in normoxia (Fig. 1 b). Additionally, the changes in the area occurred more rapidly and were sustained over the course of the experiment in hypoxic cells, whereas the area of normoxic cells quickly returned to baseline or decreased (Fig. 1 b). As cell area is influenced by both protrusion and retraction events, we utilized the publicly available code from the Danuser lab (Lee et al., 2015) to more accurately quantify cellular protrusions. In this approach, the algorithm traces individual cells, assigns unbiased sampling windows along the edge of the cell (programmed default is 10 μm wide), and determines protrusion vectors based on changes in the location of the cell edge between each frame. The resulting data were quantified as the protrusive velocity over time for each cell and displayed as a 3D heatmap. To determine whether hypoxia altered the basal frequency and amplitude of protrusion events, DU145-GFP-Lifeact cells were cultured overnight in normoxia or hypoxia, then grown in starvation media for 4 h, and imaged every 15 s. Strikingly, hypoxic cells displayed significantly more frequent and intense protrusive events under basal conditions than normoxic cells (Fig. 1 c). Quantification of these protrusion vectors revealed a significant increase in the protrusive activity of DU145 cells in hypoxia. Similar results were observed in MCF7 breast cancer cells, indicating that the effect of hypoxia on protrusive activity is not specific to PCa cells (Fig. 1 d). Taken together, our findings indicate that hypoxia increases the basal levels of membrane protrusion and primes cancer cells to respond to growth factors.

Previous work in our lab demonstrated that PIM1 is upregulated in hypoxia, independent of HIF-1 signaling (Chen et al., 2009; Warfel and Kraft, 2015), and treatment with a small-molecule PIM inhibitor reduces tumor metastasis in orthotopic models of prostate and colon cancer (Casillas et al., 2018). Based on these findings, we reasoned that PIM1 may play an important role in mediating the proinvasive effect of hypoxia. To test this hypothesis, we implemented a 3D invasion assay where PC3-LN4 or PC3-LN4 cells lacking PIM1 (CRISPR knockout) were seeded into Matrigel as part of an inner core surrounded by a collagen matrix. Then, the chambers were cultured in normoxia (20% O_2_) or hypoxia (1% O_2_; PIM1 levels are shown in Fig. S1 b), and tumor cells were allowed to invade the outer matrix. After 48 h, the cells were fixed and stained for DNA. The number of invading cells and the distance from the edge of the core to each nucleus were measured (Fig. 1 e). Strikingly, the number of invading cells was significantly reduced in cells lacking PIM1 (Fig. 1 f). The fold-change decrease in invasion depth (11.2-fold decrease) observed in PIM1-KO cells was identical in normoxia and hypoxia, suggesting that basal levels of PIM1 are important for invasion, regardless of oxygen tension. The average distance of invasion was significantly increased in hypoxia compared with normoxia, and this effect was significantly blunted in PC3-LN4 cells lacking PIM1 (Fig. 1 g). There was also a significant increase in invasion in the PIM1-KO cells in hypoxia; this could be evidence that upregulation of PIM2/3 in hypoxia partially rescues this phenotype. To confirm that PIM activation is critical for hypoxia-induced invasion, we performed Boyden chamber invasion assays using PC3-LN4 cells. As expected, hypoxia significantly increased the number of invading cells compared with normoxia. Strikingly, treatment with a PIM inhibitor (AZD1208) blocked the increase in invasion seen in hypoxia, providing further evidence that PIM activity is required for the proinvasive effect of hypoxia (Fig. S1 a). Next, we assessed the role of PIM1 in cell migration by live cell microscopy using PC3-LN4 cells with PIM1 overexpression or CRISPR knockout. Each cell line was plated sparsely and conditioned in normoxia or hypoxia for 4 h, and the distance traveled by single cells over a 24-h time course was measured. Both exposure to hypoxia and overexpression of PIM1 significantly increased the average distance traveled by each cell (Fig. S1 c). Notably, hypoxia did not further increase motility in cells overexpressing PIM1. Importantly, knockout of PIM1 reduced migration compared with that in control cells in normoxia, and loss of PIM1 completely negated the hypoxia-induced increase in cell migration (Fig. S1 b). Wound healing assays using mouse embryonic fibroblasts (MEFs) harboring wild-type PIM (WT) or total knockout (TKO) of all three PIM isoforms further demonstrated the necessity of PIM signaling for proper migration (Fig. S1, d and e). To further establish the importance of PIM in regulating protrusion dynamics, we measured spreading area over time as a dynamic measurement of leading-edge protrusion. During cell spreading, actin dynamics play a significant role in pushing the leading edge, resulting in protrusion and cell spreading. WT and TKO MEFs were plated on laminin-coated dishes in serum-free media, and images were taken every 1 min for 2 h. The area of individual cells (*n* = 20/cell line) was measured to determine the rate of cell spreading. Within 90 min, WT MEFs reached the maximum area (∼3,000 μm^2^), whereas spreading was significantly slower in TKO MEFs, only reaching ∼1,700 μm^2^ by 120 min (Fig. S1 d). Together these data demonstrate that PIM plays an important role in regulating cellular protrusion and motility, particularly in hypoxia.

### ABI2 is a direct substrate of PIM kinase

**Figure 2.**
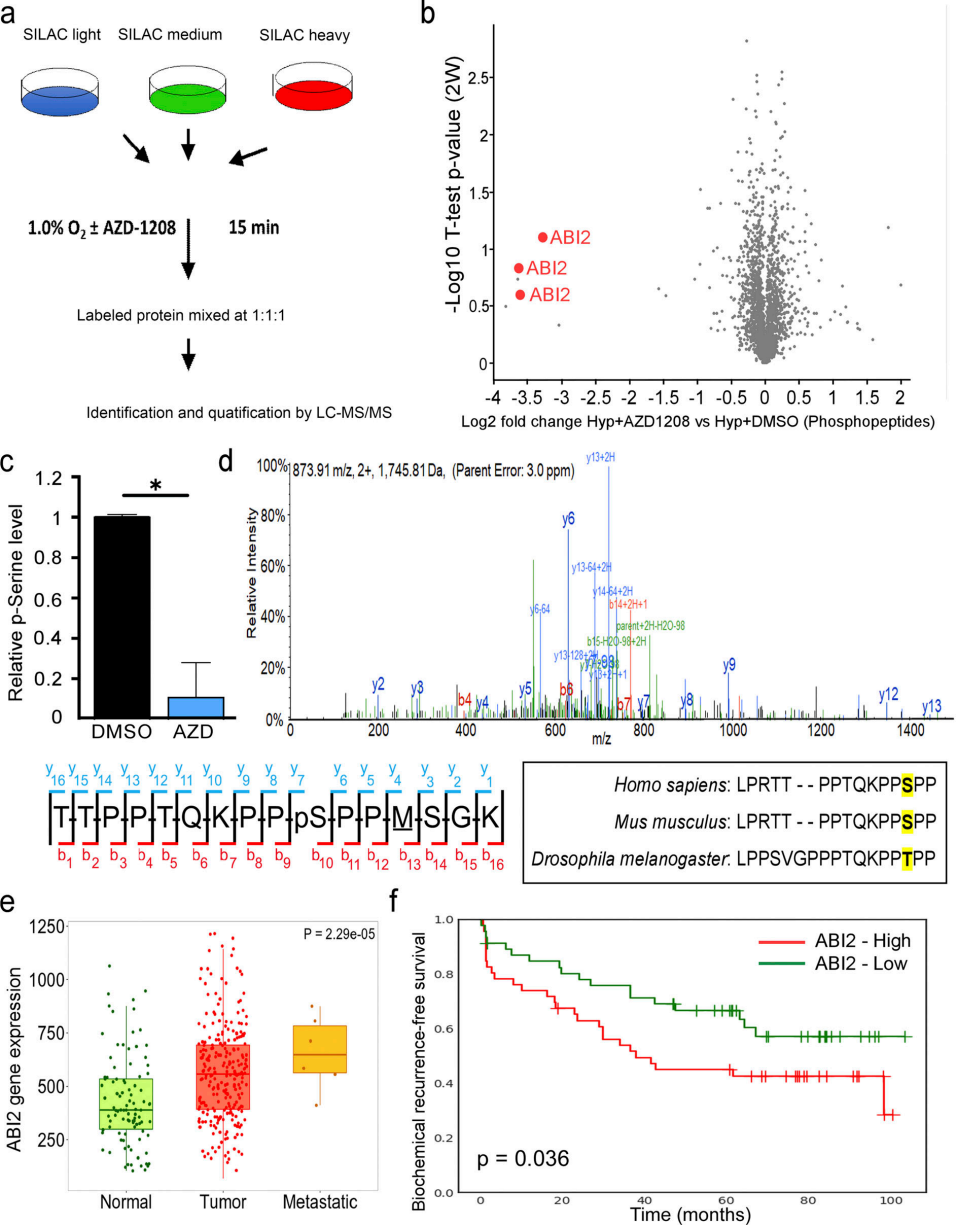
**Identification of ABI2 as a novel PIM substrate. (a)** Experimental strategy for the identification of novel PIM substrates using SILAC. **(b)** Volcano plot of the phosphopeptides that were significantly increased and decreased, as determined by a one-sample *t* test in hypoxia after AZD1208 treatment. **(c)** Graph depicting relative serine phosphorylation of peptides containing Ser183 in the presence and absence of PIM inhibition. Error displayed as 95% CI as determined by one-sided Student’s *t* test (*P < 0.05). **(d)** Spectra from mass spectrometry analysis of ABI2 from in vivo kinase assay showing phosphorylation of Ser183 by PIM1. Inset = sequence alignment across species of ABI2 Ser183 (highlighted). **(e)** *ABI2* gene expression in human prostate tumor samples from the National Center for Biotechnology Information GEO database (TNMplot web analyzer). **(f)** Kaplan–Meier analysis of biochemical recurrence-free survival in PCa patient cohorts based on the expression of ABI2.

**Figure S2.**
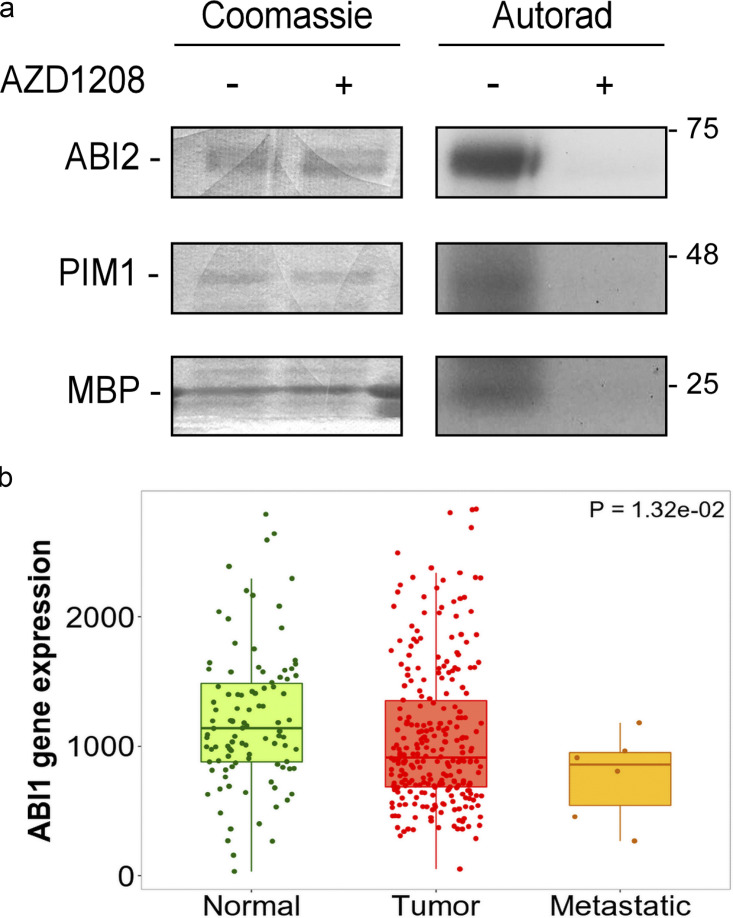
**PIM1 phosphorylates ABI2 in vitro. (a)** Coomassie and autoradiography of in vitro kinase assay using recombinant ABI2, PIM1, and MBP (positive control) in the presence or absence of PIM inhibitor AZD1208. **(b)** *ABI1* gene expression in human prostate tumor samples from the National Center for Biotechnology Information Geo database (TNMplot web analyzer).

**Figure 3.**
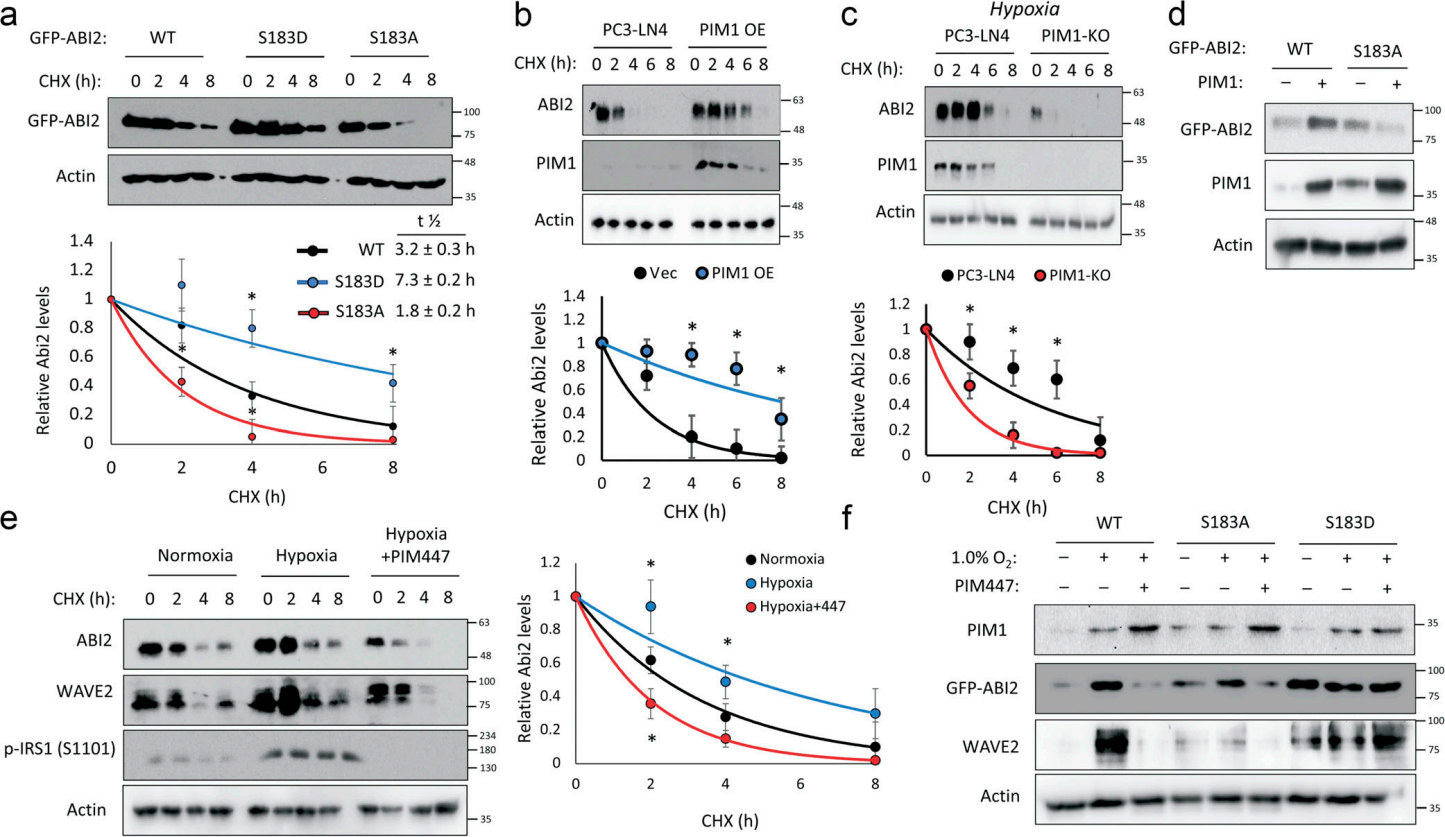
**Phosphorylation at Ser183 by PIM1 increases ABI2 stability. (a)** 293T cells were transfected with GFP-ABI2 WT, S183A, or S183D prior to treatment with cycloheximide (CHX, 10 μM), and GFP levels were assessed by Western blotting. Densitometry was used to determine the rate of protein decay. **(b and c)** PC3-LN4 cells transfected with control vector or human PIM1 under normoxia (c) PC3-LN4 and PIM1-KO cells were treated with CHX for the indicated times at 1% O_2_, and ABI2 levels were assessed by Western blotting. **(d)** PC3-dox inducible PIM1 cells were transfected with GFP-tagged WT-ABI2 or GFP-tagged S183A-ABI2 and treated with DMSO (−) or dox (+) (100 ng/ml) for 24 h. Protein levels were measured by Western blotting. **(e)** DU145 cells were incubated in normoxia or hypoxia (1.0% O_2_) for 4 h prior to treatment. DMSO or PIM447 was added 30 min prior to addition of CHX, and cells were lysed at the indicated time points. **(f)** DU145 cells were transfected with GFP-ABI2 WT, S183A, and S183D under normoxic and hypoxic conditions in the presence or absence of PIM447. *P < 0.05, two-sided Student’s *t* test, *n* = 3 for all experiments, and error bars = SEM. Source data are available for this figure: SourceData F3.

Although an extensive list of PIM substrates has been described (Warfel and Kraft, 2015), none directly link PIM1 to regulation of the actin cytoskeleton. Therefore, we performed an unbiased proteomics screen incorporating stable isotope labeling of amino acids in cell culture (SILAC) to identify new PIM substrates, particularly in hypoxia. PC3-LN4 PCa cells were grown subconfluently with isotopically distinct forms of arginine. After validating SILAC incorporation, cells were conditioned in normoxia or hypoxia for 8 h and then treated with AZD1208 (3 µM) or DMSO for 15 min. This time point was chosen to capture potential direct substrates of PIM kinases as this was the earliest time point at which we observed loss of phosphorylation of eIF4B (Ser406), a known PIM1 substrate. Biological replicates were performed and protein from each treatment group was combined and processed for mass spectrometric analysis. Phosphopeptides were isolated, and liquid chromatography-mass spectrometry/mass spectrometry (LC-MS/MS) was used to identify potential PIM targets (Fig. 2 a). We identified 75 phosphopeptides that were significantly reduced in hypoxia after the addition of AZD1208 (data publicly available in PRIDE database). Notably, several of these 75 peptides belonged to microtubule and actin-related proteins capable of cytoskeletal regulation, including Actin Binding LIM Protein Family Member 3, Cytoplasmic Linker Associated Protein 1, and Cortactin. Interestingly, the most significantly downregulated phosphopeptides upon inhibition of PIM in hypoxia belonged to ABI2 (Fig. 2 b). ABI2 is an integral member of the WRC, which is responsible for recruiting ARP to the cell membrane, where it facilitates actin nucleation and the subsequent formation of lamellipodia. Specifically, we identified several peptides between Ile147 and Pro185 in ABI2 with differential phosphorylation upon PIM inhibition in hypoxia. To validate that ABI2 is a direct substrate of PIM1, we performed in vitro kinase assays. Recombinant ABI2 and PIM1 proteins were incubated along with Glutathione S-transferase (GST; negative control) and Myelin basic protein (MBP; positive control) in the presence or absence of a pan-PIM kinase inhibitor (AZD1208). Autoradiography readily demonstrated that ABI2 was phosphorylated in the presence of PIM1, and inhibition of PIM completely abrogated phosphorylation of ABI2 (Fig. S2 a). Subsequent quantitative mass spectrometry analysis revealed a dually phosphorylated peptide containing Ser183 and Ser187 in ABI2. Notably, phosphorylation of this peptide was almost completely lost upon the addition of AZD1208 (Fig. 2 c). To further confirm which site(s) are phosphorylated by PIM1, we performed cell kinase assays using immunoprecipitated ABI2 from cells and mixed with GST-PIM1 or GST alone. Mass spectrometry analysis identified Ser183 as the most consistently phosphorylated PIM1 site both in vitro and in vivo (Fig. 3 d; spectra from in vivo analysis). Sequence alignment of ABI2 indicated that S183 is conserved among species, suggesting the functional relevance of this residue (Fig. 2 d). Indicative of the importance of ABI2 in PCa metastasis, analysis of data from the National Center for Biotechnology Information Gene Expression Omnibus database (Bartha and Győrffy, 2021) showed that *ABI2* expression is significantly higher in prostate tumors than in normal tissue and it is further upregulated in metastatic castrate-resistant PCa (Fig. 2 e). Notably, the expression of an ABI2 homolog, ABI1, was not correlated with PCa progression, suggesting that they are not compensatory (Fig. S2 b). Further analysis of ABI2 in PCa using the Prostate Cancer Transcriptome Atlas (PCTA; You et al., 2016) revealed an inverse correlation between ABI2 expression and biochemical recurrence-free survival (Fig. 2 f). These data identify ABI2 as a novel substrate of PIM kinases and provide a potential link connecting PIM activity to the regulation of the actin cytoskeleton.

### Phosphorylation of ABI2 by PIM1 promotes its stability

Next, we tested the effect of PIM-mediated phosphorylation at Ser183 on ABI2. To this end, we generated phospho-mimetic (S183D) and phospho-null (S183A) mutants of ABI2. Then, WT and phospho-mutant ABI2 constructs were transiently transfected into 293T cells, and cycloheximide chases were performed to assess differences in ABI2 protein stability. Densitometry analysis of Western blotting revealed that the S183D (half-life = 7.3 ± 0.2 h) and S183A (half-life = 1.8 ± 0.2 h) mutants were significantly more and less stable, respectively, than WT ABI2 (half-life = 3.2 ± 0.3 h; Fig. 3 a). To determine whether PIM1 controls ABI2 protein stability, cycloheximide chase experiments were performed in the previously described PC3-LN4 cell model with stable PIM1 overexpression or knockout. In normoxia, overexpression of PIM1 significantly increased the half-life of ABI2 compared with control (half-life = 7.6 ± 0.3 vs. 3.1 ± 0.2 h, P < 0.05; Fig. 3 b). Preconditioning in hypoxia significantly increased ABI2 stability (half-life = 4.6 ± 0.2 h, P < 0.05) compared with vector control in normoxia (Fig. 3 c), and PIM1 knockout significantly reduced ABI2 stability (half-life = 2.0 ± 0.1 h, P < 0.05) compared with control in hypoxia (Fig. 3 c). To test whether phosphorylation at Ser183 is required for PIM1 to stabilize ABI2, we overexpressed GFP-ABI2 WT or the S183A mutant in DU145 cells stably expressing vector or PIM1, and ABI2 levels were monitored by Western blotting. We found that PIM1 overexpression increased WT ABI2, whereas the S183A mutant was refractory to PIM1 overexpression (Fig. 3 d), suggesting that phosphorylation of S183 is required for PIM1 to increase ABI2 levels. Lastly, to determine whether the upregulation of ABI2 in hypoxia requires PIM1 activity, we performed cycloheximide chase experiments in DU145 cells in the presence or absence of PIM447. Hypoxia increased the stability of ABI2 compared with normoxia (half-life = 4.4 ± 0.2 vs. 2.9 ± 0.1 h), whereas inhibition of PIM in hypoxia reduced ABI2 protein stability below that observed in normoxia (half-life = 1.7 ± 0.2 h; Fig. 3 e). Interestingly, another member of the WRC, WAVE2, displayed similar kinetics to ABI2, showing increased stability in hypoxia that was negated by PIM447 treatment (Fig. 3 e). To confirm that hypoxia induced WAVE2 levels via activation of PIM1 and subsequent phosphorylation of ABI2 S183, DU145 cells were transfected with GFP-ABI2 (WT) or ABI2 mutants (S183A or S183D). After transfection, cells were cultured in normoxia or hypoxia in the presence or absence of PIM447. Hypoxia markedly increased both ABI2 and WAVE2 in the cells overexpressing WT ABI2. In contrast, WAVE2 levels did not respond to hypoxia or PIM inhibition in the cells expressing mutant ABI2 (Fig. 3 f). Moreover, basal levels of ABI2 S183A and S183D mutants were noticeably lower or higher, respectively, compared with WT. These data demonstrate that upregulation of PIM1 in hypoxia increases ABI2 levels via direct phosphorylation of Ser183.

### PIM1 promotes WRC formation and activation in an ABI2-dependent manner

**Figure 4.**
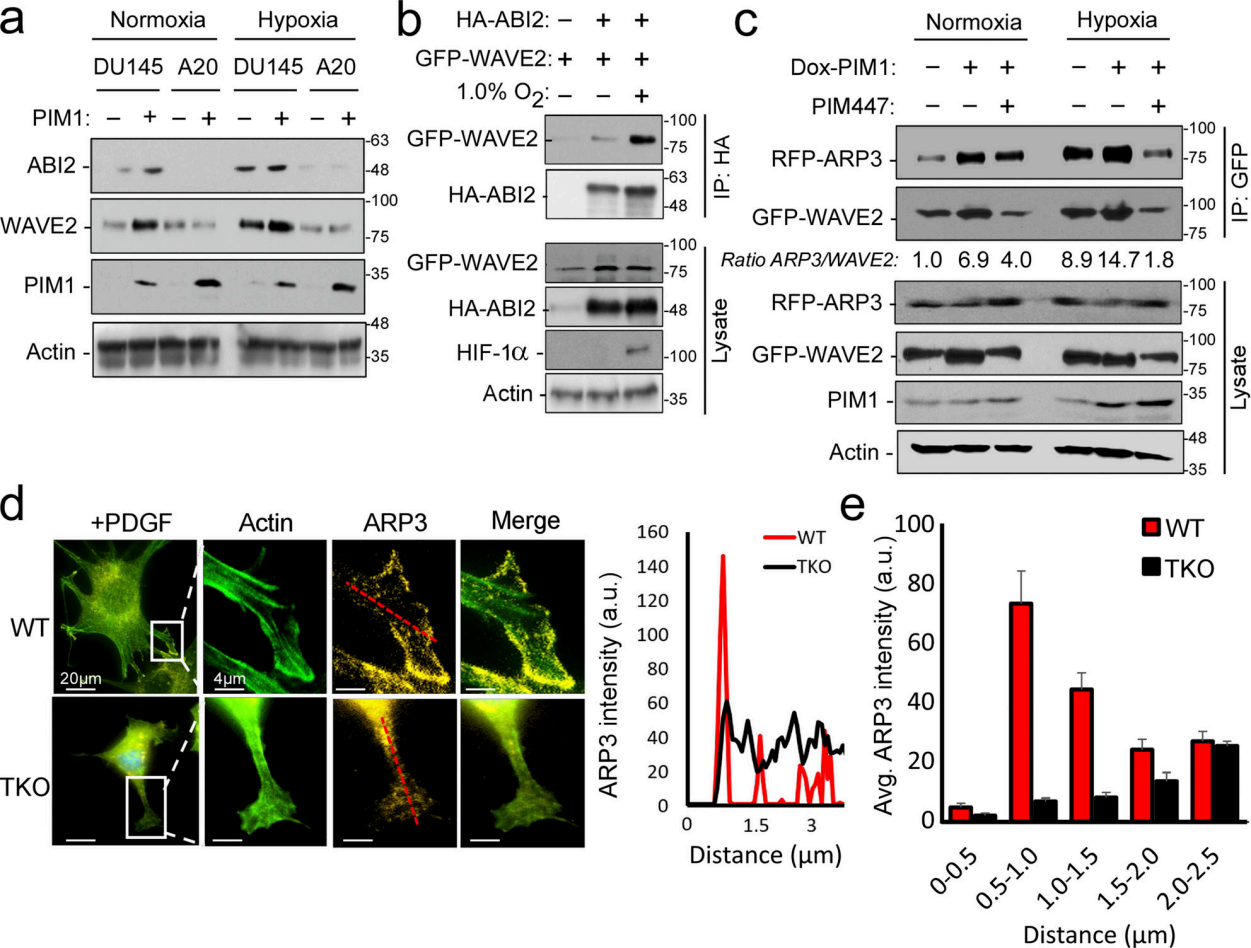
**Hypoxia and PIM1 increase WRC levels and activation. (a)** DU145 or ABI2-KO_A20_ cells stably expressing vector or PIM1 were cultured in normoxia or hypoxia (1.0% O_2_) for 16 h, and Western blotting was used to assess protein levels. **(b)** 293T cells were transfected with HA-ABI2 and GFP-WAVE2 and cultured in normoxia or hypoxia for 4 h. ABI2 was immunoprecipitated using anti-HA beads, and WAVE2 binding was assessed by Western blotting for GFP. **(c)** PC3-dox PIM1 cells were transfected with GFP-WAVE2 and RFP-ARP3. Then, cells were placed in normoxia or hypoxia and treated with dox (100 ng/ml) for 4 h in the presence of DMSO or PIM447 (3 µM). 5 min prior to harvest, all samples were stimulated with EGF (50 nM). WAVE2 was immunoprecipitated using the GFP tag, and ARP3 binding was assessed by Western blotting for RFP. **(d)** WT or TKO MEFs stably expressing GFP-Lifeact and RFP-ARP3 were starved for 4 h prior to stimulation with PDGF for 15 min. Cells were fixed for imaging, and ARP3 intensity was measured from the edge of cellular protrusions toward the center of the cell body (red dotted line). **(e)** Quantification of ARP3 intensity at given increments of distance from the leading edge (*n* > 15 cells/group). * P < 0.05 using two-sided Student’s *t* test, error = SEM. Scale bar = 20 µm for widefield images and 4 µm for insets. Source data are available for this figure: SourceData F4.

**Figure S3.**
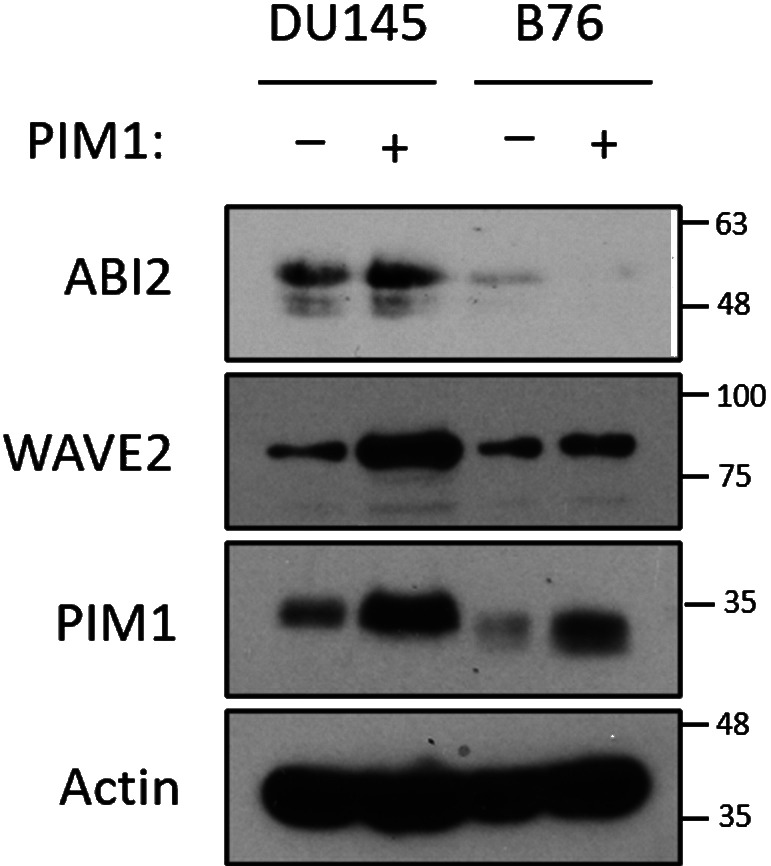
**PIM1 increases WAVE2 levels in a ABI2-dependent manner.** DU145 or ABI2-KO_B76_ cells stably expressing vector or PIM1 were cultured in normoxia for 16 h, and Western blotting was used to assess protein levels. Source data are available for this figure: SourceData FS3.

Previous reports suggest that the expression level of any single component of the WRC could impact the stability of other WRC members (Rottner et al., 2021). Therefore, we hypothesized that PIM1-mediated stabilization of ABI2 in hypoxia could increase the amount of the WRC. Our previous results show that hypoxia increased WAVE2 levels in a PIM-dependent manner (Fig. 3 e). To determine whether the upregulation of WAVE2 by PIM1 was dependent on ABI2, we generated two DU145 cell lines with CRISPR-knockout of ABI2 (ABI2-KO_A20_ and ABI2-KO_B76_). DU145 and ABI2-KO_A20_ cells with or without stable overexpression of PIM1 were cultured in normoxia and hypoxia, and the expression of ABI2 and WAVE2 was assessed by Western blotting. Hypoxia increased WAVE2 in DU145 but not ABI2-KO_A20_ cells (Fig. 4 a). Notably, overexpression of PIM1 increased WAVE2 levels in DU145 cells in both normoxia and hypoxia, whereas no change was observed in ABI2-KO_A20_ cells in either condition (Fig. 4 a). These results were confirmed in ABI2-KO_B76_ cells (Fig. S3). These data indicate that ABI2 is required for PIM1 to increase WAVE2 levels. Next, we asked whether hypoxia increased the relative levels of ABI2 bound to WAVE2, suggesting the formation of the WRC. To this end, HA-ABI2 and GFP-WAVE2 were transfected into DU145 cells cultured in normoxia or hypoxia for 16 h. WAVE2 was immunoprecipitated using the GFP tag, and ABI2 binding was assessed by immunoblotting for HA. Both ABI2 and WAVE2 levels were increased by hypoxia and the total amount of WAVE2 that co-immunoprecipitated with ABI2 under hypoxic conditions was greater than that from cells cultured in normoxia (Fig. 4 b).

Activation of the WRC and the initiation of actin branching occurs upon WAVE2 binding to ARP2/3. Therefore, we used co-immunoprecipitation as a readout of relative WRC activation. GFP-WAVE2 and RFP-ARP3 were overexpressed in PC3-LN4 cells stably expressing dox-inducible PIM1. Following transfection, cells were treated with doxycycline and/or PIM447 for 24 h to induce or inactivate PIM1, respectively. Hypoxia increased the relative amount of ARP3 pulled down with WAVE2 (Fig. 4 c, lane 1 vs. 4). Overexpression of PIM1 further increased WRC activation in both normoxia and hypoxia, and PIM inhibition blocked the increased association between WAVE2 and ARP3, particularly in hypoxia (Fig. 4 c).

Another indicator of WRC activation is the localization of ARP3 to the leading edge of cellular protrusions. Hence, we next investigated whether loss of PIM altered the localization of ARP2/3 in response to growth factor stimulation. WT and TKO MEFs were stably transduced with GFP-Lifeact (F-actin) and RFP-ARP3. Cells were then serum-starved and placed in hypoxia for 4 h prior to the addition of platelet-derived growth factor (PDGF) for 30 min to stimulate ARP2/3 activation. Upon stimulation, ARP3 was highly localized to sites of actin nucleation (the leading edge of lamellipodia) in WT MEFs, whereas ARP3 remained evenly distributed throughout the cell in TKO MEFs (Fig. 4 d). Representative line scans of lamellipodia indicate that ARP3 intensity spiked at the leading edge in WT MEFs compared with the remaining cell body. In contrast, there was no significant difference in ARP3 intensity between the leading edge and cell body in TKO MEFs (Fig. 4 d). Quantification of ARP3 fluorescence intensity in 0.5-μm increments across multiple cells (*n* > 15) demonstrates that ARP3 localization to the leading edge was deficient in TKO MEFs compared with WT MEFs (Fig. 4 e). Taken together, our results indicate that PIM1 increases WRC levels and promotes ARP activation at the leading edge of PCa cells.

### ABI2 is essential for hypoxia and PIM1-driven protrusions

**Figure 5.**
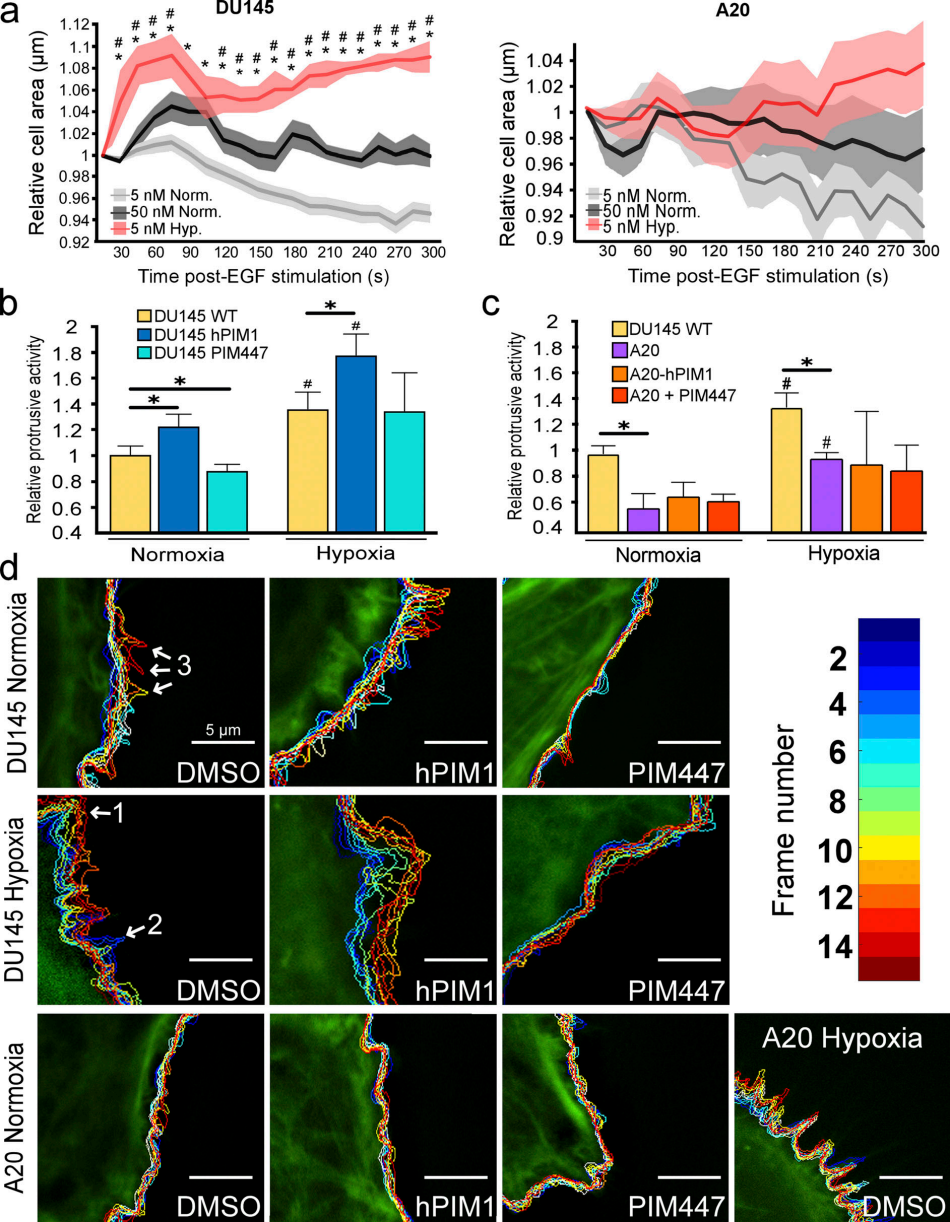
**ABI2 is necessary for hypoxia- and PIM-induced protrusions. (a)** DU145 and ABI2-KO_A20_ cells were stimulated with the indicated doses of EGF in normoxia or hypoxia, and cell area was plotted relative to the pre-stimulated average area. The shaded area represents 95% CI as determined by two-sided Student’s *t* test (*P < 0.05 between Hyp. 5 nM and Norm. 5 nM and *P < 0.05 between Hyp. 5 nM and Norm. 50 nM; *n* = 6). **(b)** DU145 cells were imaged in normoxia and hypoxia in the presence or absence of PIM447 (3 µM). The relative proportion of randomly sampled windows in DU145 or DU145-PIM1-overexpressing cells actively protruding during progressive frames (15-s intervals): DU145 WT (*n* = 1,637, *N* = 12), DU145-hPIM1 (*n* = 1,395, *N* = 12), and DU145-PIM447 (*n* = 1,682, *N* = 11). In hypoxia, DU145 WT (*n* = 1,268, *N* = 5), DU145-hPIM1 (*n* = 1,333, *N* = 10), and DU145-PIM447 (*n* = 2,056, *N* = 10). Error is shown as 95% CI as determined by two-sided Student’s *t* test (*P < 0.05, *P < 0.05 between normoxic and hypoxic conditions within each cell line). **(c)** Quantification of unstimulated cell protrusive activity in ABI2-KO_A20_ (*n* = 2,116 Nx, *N* = 9 and *n* = 1,279 Hx, *N* = 6), ABI2-KO_A20_-hPIM1 (*n* = 1,213 Nx, *N* = 7 and *n* = 2,183 Hx, *N* = 10), and ABI2-KO_A20_-PIM447 (*n* = 1,899 Nx, *N* = 10 and *n* = 2,133 Hx, *N* = 10) cells. Error = 95% CI as determined by two-sided Student’s *t* test (*P < 0.05, *P < 0.05 between normoxic and hypoxic conditions within each cell line). **(d)** Representative cell traces depicting changes in actin structure over time in DU145 and ABI2-KO_A20_ cells overexpressing PIM1 or treated with PIM447 under normoxic and hypoxic conditions. Regions representative of changes in actin structure are marked as follows 1 = lamellipodia, 2 = retraction, 3 = transient protrusion. Scale bar = 5 µm for all images.

**Figure S4.**
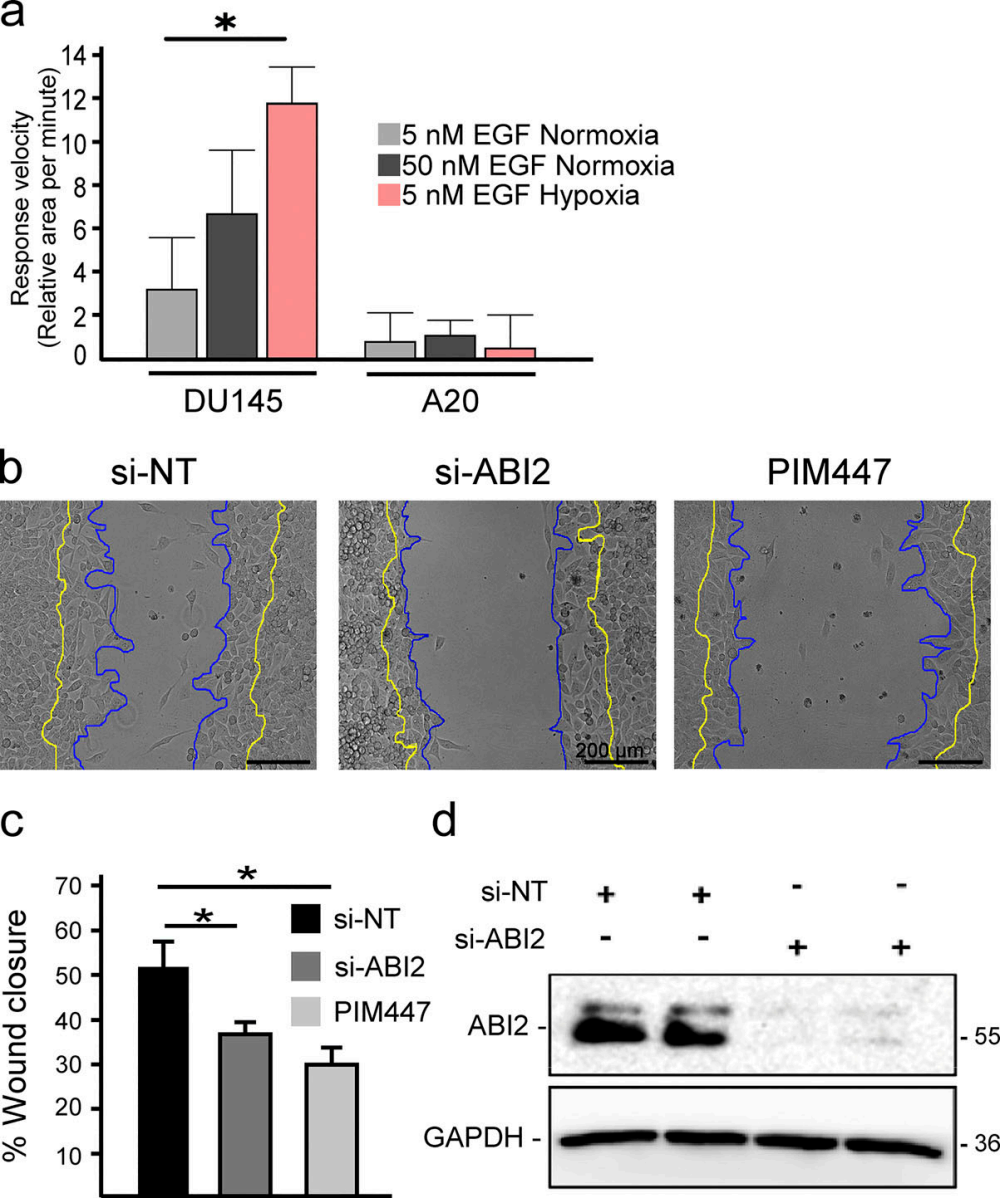
**ABI2 and PIM are necessary for proper wound healing. (a)** Quantification of the change in cell area per minute upon stimulation with EGF in different oxygen environments. Error = 95% CI (*n* = 45 5 nM EGF Nx; *n* = 15, 50 nM EGF Nx; and *n* = 15, 5 nM EGF Hx for DU145; *n* = 6 for ABI2-KO_A20_ in all conditions; *P < 0.05). **(b)** Wound healing assay with PC3-LN4 cells with a non-targeting siRNA, ABI2-targeting siRNA, or chemical inhibition of PIM. Cell edge at the time of scratch depicted in yellow and edge 8 h post-scratch in blue. Scale bar = 200 µm. **(c)** Quantification of the scratch assay. Error is SEM (*n* = 3; *P < 0.05). **(d)** Western blot depicting efficacy of siRNAs targeting ABI2 or non-specific sequence (negative control). Source data are available for this figure: SourceData FS4.

Based on our biochemical data showing more WRC formation in hypoxia, we reasoned that hypoxia may sensitize cells to chemotaxis. To test this, DU145 cells were stimulated with low (5 nM) and high (50 nM) concentrations of EGF. In normoxia, stimulation with 5 nM EGF did not significantly increase cell area, whereas 50 nM EGF caused a rapid and pronounced response (Fig. 5 a). Strikingly, treatment with a low dose of EGF (5 nM) significantly increased the speed and amplitude of protrusions in hypoxic cells to a greater extent than that observed in normoxic cells stimulated with a high dose of EGF (Fig. 5 a). Next, we asked whether ABI2 is required for the hypoxia-induced sensitization to growth factor stimulation. To this end, we measured changes in cell area following stimulation with low or high doses of EGF in DU145 and ABI2-KO_A20_ cells conditioned in normoxia and hypoxia. In contrast to DU145 cells, ABI2-KO_A20_ cells did not respond to either a low or high concentration of EGF, regardless of oxygen concentration (Fig. S4 a). To confirm the effect of ABI2 loss on cell migration, we performed wound healing assays using a non-targeting siRNA (si-NT), si-ABI2, or si-NT + PIM447. Knockdown of ABI2 and chemical inhibition of PIM significantly reduced wound closure to the same extent compared with controls (Fig. S4, b–d).

Next, we assessed the impact of PIM1 on protrusive activity. PIM1 was stably overexpressed in DU145-GFP-Lifeact cells, and basal protrusive activity, lacking growth factor stimulation, was measured as described previously. Overexpression of PIM1 significantly increased cellular basal levels of protrusion in normoxia compared with empty vector controls (Fig. 5 b). Contrastingly, treatment with PIM447 significantly decreased protrusive activity (Fig. 5 b). Exposure to hypoxia significantly increased the protrusive activity of both control and PIM1-overexpressing cells, and treatment with PIM447 significantly blunted the ability of hypoxia to enhance protrusive activity (Fig. 5 b). We then asked whether the increased protrusive activity resulting from hypoxia and PIM1 requires ABI2. DU145 and ABI2-KO_A20_ cells were stably transfected with vector or PIM1 and cultured in hypoxia for 16 h prior to the addition of DMSO or PIM447. Loss of ABI2 blunted the increase in protrusive activity observed with hypoxia and overexpression of PIM1 (Fig. 5 c). To better understand how hypoxia alters membrane dynamics, we superimposed the cell outlines at each timeframe onto a single image as a function of time. We observed evidence of prolonged lamellipodia-like protrusions (Fig. 5 d, noted “1”), retractions (Fig. 5 d, noted “2”), and other short-lived protrusions (Fig. 5 d, noted “3”) that were more transient in nature, with the last type being most common in normoxia. In contrast, hypoxic cells displayed larger and more dynamic protrusion events, as evidenced by larger lamellipodia-like protrusions that extended further away from the cell body than those occurring in normoxia. Overexpression of PIM1 was sufficient to increase the frequency and extent of protrusions in normoxia and further increased protrusive activity under hypoxia. Treatment with PIM447 significantly decreased protrusive activity in both normoxia and hypoxia with a striking loss of almost all protrusions in normoxia. Cell edge traces of ABI2-KO_A20_ cells show very little movement compared with that of DU145 cells in all conditions. Surprisingly, hypoxia was still able to increase the protrusive activity of ABI2-KO_A20_ cells compared with normoxia (Fig. 5 c). However, the protrusive phenotype was drastically different in cells lacking ABI2 (Fig. 5 d). DU145 cells displayed broad lamellipodia that are typical of WRC-mediated protrusions, whereas ABI2-KO_A20_ cells produced rapid, spike-like protrusions that are typical of filopodia (Fig. 5 d). These data demonstrate that PIM1 and hypoxia drive actin dynamics and lamellipodia formation through the stabilization of ABI2.

### PIM inhibition blocks PCa invasion in vivo

**Figure 6.**
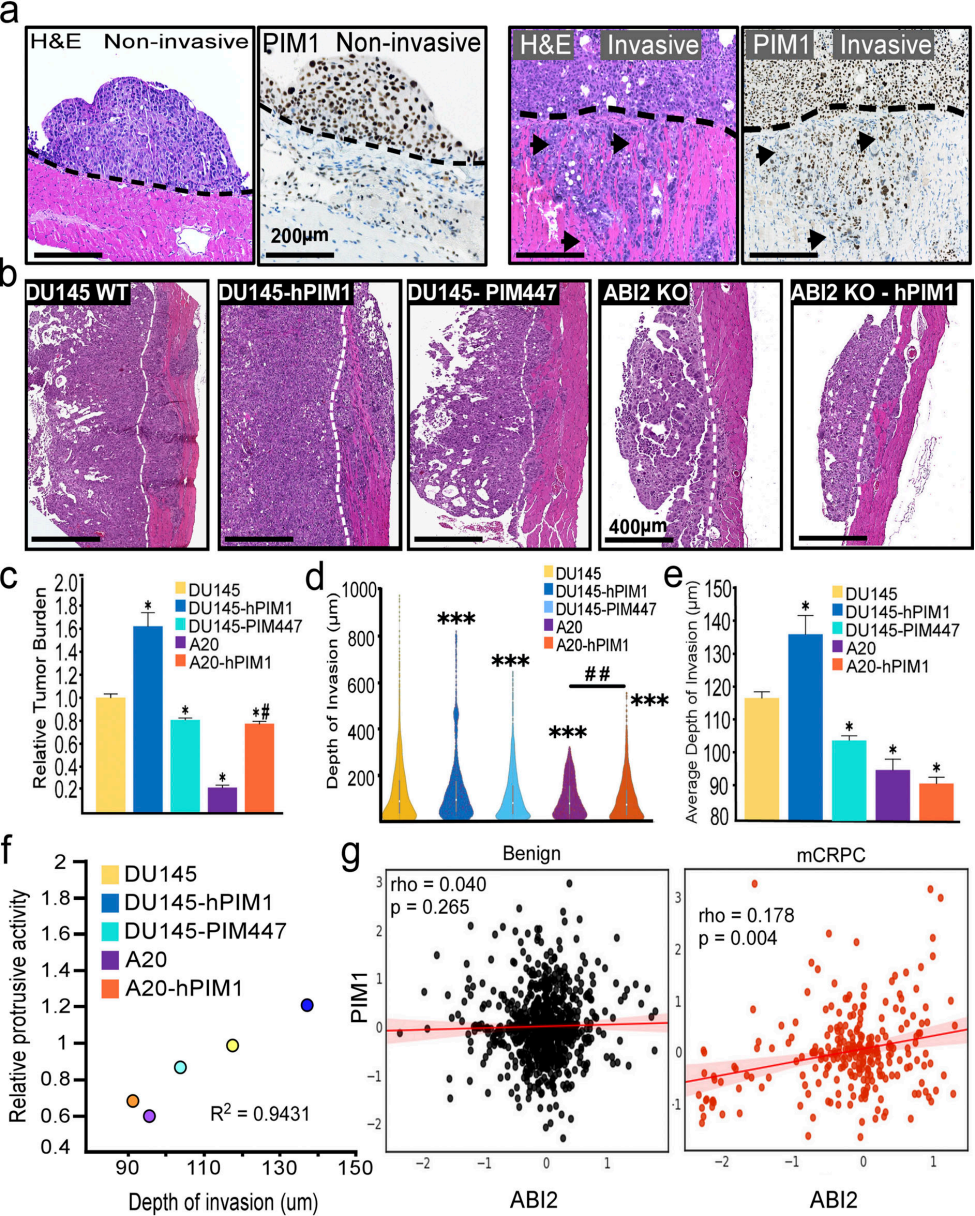
**PIM1-ABI2 signaling controls tumor cell invasion in vivo. (a)** Representative images of PIM1 expression in non-invasive and invasive tumor seeded on the diaphragm (black dotted line indicates the tumor/diaphragm interface). **(b)** Representative images of DU145 and ABI2-KO_A20_ tumors with PIM1 overexpression or PIM447 treatment (white dotted line indicates the tumor/diaphragm interface). **(c)** Relative tumor burden was quantified based on H&E staining of the total tumor area compared with parental DU145 tumors (*n* = 12, *N* = 3). Error = 95% CI as determined by two-sided Student’s *t* test (*P < 0.05 compared with DU145 vector, *P < 0.05 compared with ABI2-KO_A20_ vector). **(d)** Depth of invasion from the tumor/diaphragm interface of individual tumor cells was measured and a Kolmogorov–Smirnov test was run to determine the statistical difference among the distributions (***P < 0.001 as compared with DU145-DMSO treated cells and **P < 0.01 between A20-DSMO and A20-hPIM1 cells). **(e)** The average depth of invasion was graphed for DU145 (*n* = 11,951, *N* = 12), DU145-hPIM1 (*n* = 12,314, *N* = 12), DU145-PIM447 (*n* = 12,547, *N* = 12), ABI2-KO_A20_ (*n* = 1,942, *N* = 7), and ABI2-KO_A20_-hPIM1 (*n* = 5,275, *N* = 12) tumors. **(f)** Plot depicting the depth of tumor cell invasion in vivo vs. relative protrusive activity. **(g)** Gene expression data of PIM1 and ABI2 from benign and metastatic PCa patient samples.

To escape the gland, PCa cells must traverse through a layer of smooth muscle. To accurately represent the human course of PCa metastasis, we utilized an in vivo smooth muscle invasion assay (Mccandless et al., 1997; Rubenstein et al., 2019) to validate the role of the PIM1-ABI2 signaling axis in tumor invasion. First, DU145 cells (with or without stable overexpression of PIM1) were injected into the peritoneum of male severe combined immunodeficient (SCID) mice and allowed to seed onto the diaphragm for 2 wk. Then, the mice were randomly segregated into groups for treatment with vehicle or PIM447 (30 mg/kg, p.o., daily). The mice were sacrificed 4 wk after injection and the diaphragms were harvested for immunohistochemistry. Across all samples, PIM1 was strongly expressed in tumor cells seeded onto the peritoneal side of the diaphragm and expression was retained in invading cells (Fig. 6 a). We then measured tumor burden (i.e., the amount of diaphragm with tumor present on it). Mice injected with DU145-hPIM1 cells had a significantly increased tumor burden compared with mice injected with DU145-Vec or ABI2-KO_A20_-Vec cells (Fig. 6 c). Conversely, inhibition of PIM in mice injected with DU145 cells significantly reduced the tumor burden, confirming the established antitumor effect of PIM inhibitors (Fig. 6 c). Next, we quantified the average distance tumor cells invaded the diaphragm by measuring the distance from the nuclei of individual tumor cells to the thoracic tumor/diaphragm interface (Fig. 6 b; white dotted lines). DU145 cells successfully invaded the diaphragm, with an average depth of 116 µm, but few were able to invade entirely through the diaphragm muscle and into the thoracic cavity (Fig. 6 b). Overexpression of PIM1 significantly increased the average depth of invasion (135 µm, P < 0.05) and markedly increased the number of cells that fully invaded through the diaphragm (Fig. 6 b). Strikingly, treatment with PIM447 in parental cells significantly reduced the average depth of invasion (105 µM, P < 0.05) and prevented invasion through the diagram (Fig. 6 b). These data demonstrate that PIM1 enhances tumor cell invasion, and blocking PIM is sufficient to reduce the invasive potential of PCa cells.

**Figure S5.**
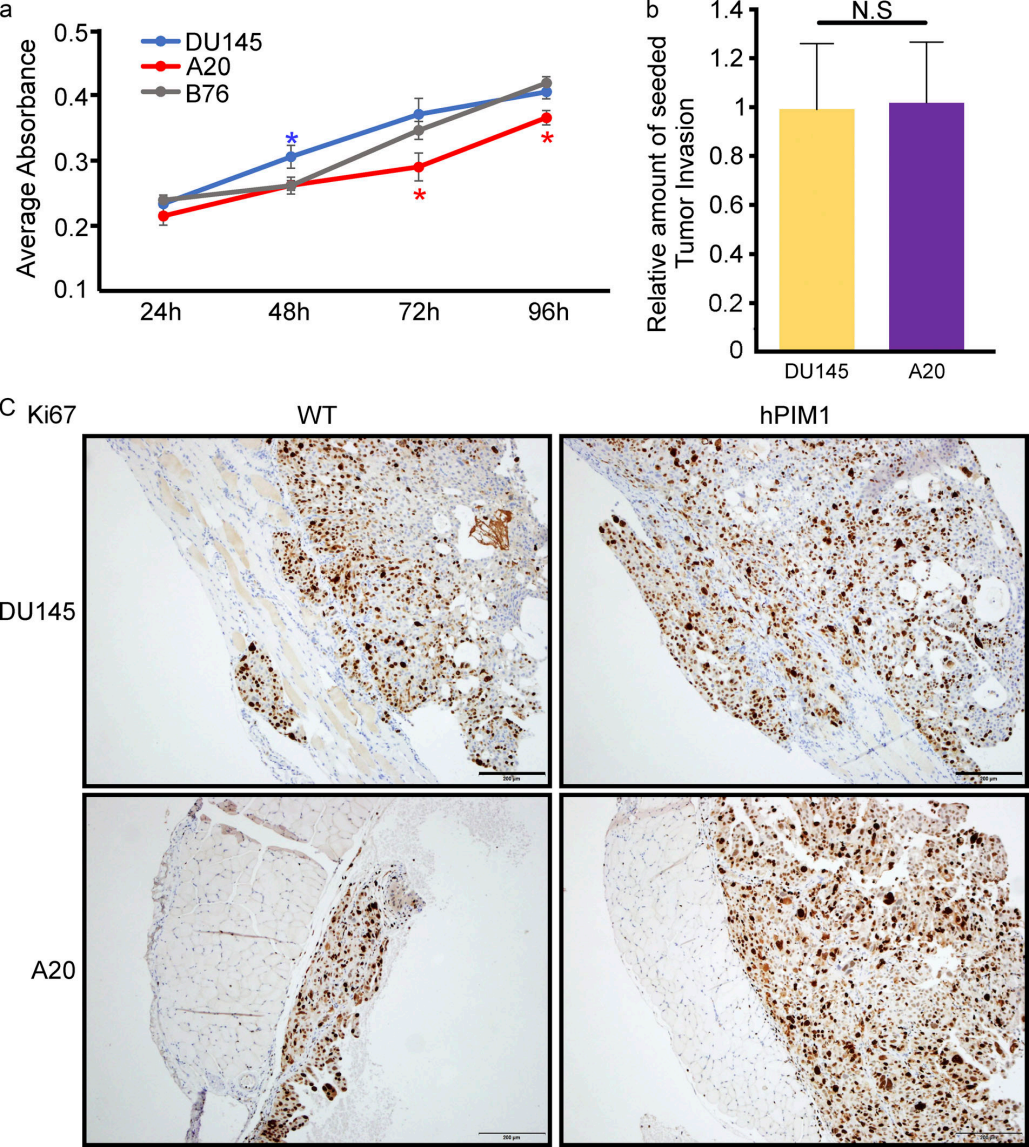
**Loss of ABI2 does not affect cell proliferation. (a)** Cells were plated in 96-well dishes and allowed to grow for 96 h. Wells were stained with Crystal Violet and eluted using SDS. Average absorbance was measured as an indirect measure of the number of cells present and plotted over time. Error is SEM (*n* = 3; *P < 0.05; color indicates point is significant compared with other cell lines at an indicated time). **(b)** Graph of relative tumor penetrance representing the proportion of seeded tumor that went on to invade through into the smooth muscle of the diaphragm of A20-DMSO treated mice (*n* = 4) in relation to DU145-DMSO treated mice (*n* = 12). Error shown as 95% CI. No significance was observed using two-sided Student’s *t* test. **(c)** IHC analysis of Ki67 expression in mouse diaphragms injected with DU145 and ABI2-KO_A20_ cells with or without overexpression of PIM1. Scale bar = 200 µm.

Next, we tested the importance of ABI2 for PIM-induced invasion using ABI2-KO_A20_ cells with or without stable overexpression of PIM1. Compared with parental DU145 cells, ABI2-KO_A20_ cells were significantly less invasive into the muscle, and we observed no invasion into the thoracic cavity (Fig. 6 b). Surprisingly, the overall volume of ABI2-KO_A20_ tumors was significantly smaller than that of DU145 tumors (Fig. 6 c). We observed a modest but significant reduction in the proliferation of ABI2-KO_A20_ cells in vitro compared with DU145 cells (Fig. S5 a), but the immunohistochemical assessment of Ki67 revealed no difference in staining between ABI2-KO_A20_ and DU145 cells (Fig. S5 c). Therefore, reduced cell growth is unlikely to account for the smaller tumor volume observed with ABI2-KO_A20_ cells. Tumor-cell interaction may explain why the loss of ABI2 impacts tumor growth, as ABI2 knockout has been implicated in reduced matrix adhesion and cell–cell adhesions (Li et al., 2007; Ryu et al., 2009). To ensure that the deficiency in invasion was not due to a decreased ability of ABI2-KO_A20_ cells to seed on the diaphragm, we assessed tumor penetrance, which indicates the proportion of seeded cells that went on to successfully invade the muscle layer. Tumor penetrance was observed to be relatively equal between DU145 and ABI2-KO_A20_ cells, indicating that tumor burden did not affect the depth of invasion (Fig. S5 b). To determine the depth of invasion, we measured the distance each nucleus invaded the smooth muscle (Fig. 6 d). Using a Kolmogorov–Smirnov test, importantly, ABI2-KO_A20_ cells also had a significantly lower average depth of invasion than DU145 cells (95 vs. 115 µm, P < 0.01), and overexpression of PIM1 in ABI2-KO_A20_ cells did not increase the depth of invasion (PIM1-overexpression vs. vector: 92 vs. 97 µm; Fig. 6 e). To determine whether increased protrusive activity resulted in more invasive tumors in vivo, we plotted the relative protrusive activity of each cell line versus the depth of invasion. This analysis showed a significant correlation between the basal rate of cellular protrusion and invasive potential in vivo (Fig. 6 f). Further data mining of the prostate cancer transcriptome atlas (PCTA) database revealed a higher correlation between PIM and ABI2 expression in metastatic castration-resistant PCa than in benign prostate tumors (Fig. 6 g). Taken together, these data demonstrate that PIM1 promotes a muscle-invasive phenotype in an ABI2-dependent manner, and inhibiting PIM significantly reduces the invasive potential of Pca cells.

## Discussion

Due to their heightened expression in a variety of solid tumors and established role in promoting angiogenesis (Casillas et al., 2018), survival (Yan et al., 2003), and cell proliferation (Wang et al., 2002), PIM kinases represent a promising anticancer target. Clinical studies demonstrate a positive correlation between PIM1 expression and tumor metastasis in breast (Gapter et al., 2006), lung (Cao et al., 2019), pancreatic (Xu et al., 2016), and prostate tumors (Santio et al., 2015). Here, we uncover a new signaling axis that poises PIM1 as a key driver of cell motility and invasion, the early steps of metastasis. This work is particularly relevant to solid tumor biology, as PIM1 is known to be increased in response to hypoxia, and our results indicate that upregulation of PIM1 is critical for hypoxia-induced invasion (Fig. 1). However, knockout of PIM1 was sufficient to significantly reduce tumor cell protrusive activity and invasion regardless of oxygen tension, suggesting that basal levels of PIM1 are important for proper cell migration. The striking decrease in the number of invaded cells and depth of 3D invasion in PIM1 KO cells suggests that PIM2 and PIM3 are not sufficient to compensate in our system (Fig 1). However, PIM2, and to a lesser extent PIM3, are also upregulated in PCa and induced by hypoxia (Warfel et al., 2016), so further experiments are warranted to determine if blocking all three PIM genes would provide an additional antimigratory effect.

Through an unbiased screening effort, we identified ABI2 as a novel substrate of PIM kinase that facilitates the regulation of cellular protrusions in both normoxia and hypoxia. In vitro and in vivo kinase assays demonstrated that PIM1 phosphorylates ABI2 at Ser183 (Fig. 2). Notably, phosphorylation of Ser183 has been observed in numerous large proteomic screens in breast, lung, and ovarian cells (Klammer et al., 2012; Mertins et al., 2014; Schweppe et al., 2013), but its functional relevance has not been reported. Interestingly, previous studies demonstrated that ABI1 can be phosphorylated at S183 in an ERK-dependent manner (Mendoza et al., 2011), and our SILAC data indicated that PIM inhibition significantly reduced ABI2 S183 phosphorylation. Thus, it is possible that S183 phosphorylation is controlled by other kinases in addition to PIM1, such as ERK, which would expand the importance of this event for the regulation of WRC activation. Phosphorylation of ABI2 has been shown to regulate its stability via ubiquitin-mediated degradation (Dai et al., 1998), but no specific sites have been identified. Ser183 is located immediately downstream of the Homeobox Homology domain of ABI2 (Dai and Pendergast, 1995; Sato et al., 2012), a region that is disordered and lacks known functional significance. ABI2 and ABI1 (a homolog) were initially identified as proteins that interact with the Abl tyrosine kinase (Dai and Pendergast, 1995). Previous studies have shown that ABI1/2 serves as a scaffold for Abl tyrosine kinase. Abl can directly phosphorylate ABI1/2 at multiple sites that differentially affect ABI1 and ABI2 protein stability, and Abl phosphorylation of WAVE2 at Y150 is sufficient to activate the WRC (Leng et al., 2005). However, Abl is unlikely to increase ABI2 protein stability or its incorporation into the WRC downstream of PIM1 because modulation of endogenous Abl activity through serum starvation, growth factor stimulation, and treatment with Abl kinase inhibitors does not alter ABI2 levels or WRC formation (Innocenti et al., 2004; Stuart et al., 2006).

Using genetic and chemical approaches, we demonstrated that phosphorylation of ABI2 at Ser183 suppresses protein turnover. Interestingly, despite the fact that Ser183 is conserved in ABI1 according to sequence alignment, ABI1 peptides were not identified in our SILAC screen as a PIM substrate in hypoxia. Moreover, ABI1 expression was decreased in PCa compared with normal prostate tissues, whereas ABI2 levels significantly increased with disease progression and predicted worse survival (Fig. 2 e). These clinical data indicate a distinct and important role for ABI2 in PCa. Our data indicate that the ability of PIM1 to stabilize ABI2 is important for the formation of the mature WRC. ABI2 is known to be critical for the stability and integrity of the WRC, and loss of ABI2 is associated with decreases in the WAVE, NAP, and SRA proteins (Dubielecka et al., 2011). However, the reverse is not well established, as overexpression of ABI2 has not been previously shown to increase the levels of other WRC members. Here, we show that the stabilization of ABI2 downstream of PIM1 increased WAVE2 protein levels (Fig. 4 a). Furthermore, PIM1 overexpression or upregulation in hypoxia increased the relative amount of ABI2 bound to WAVE2 (Fig. 4 c), suggesting that more WRC is present under these conditions. PIM1 also increased the activation of the WRC, as indicated by increased WAVE2 binding to ARP3 and localization of ARP3 to the leading edge of protrusions (Fig. 4, d and e). Importantly, the ability of PIM1 to increase WRC activity and cellular protrusion is entirely dependent on ABI2, as ABI2 knockout negated the effect of PIM1 on WRC formation and protrusion. These findings suggest a model where PIM1 upregulation in hypoxia stabilizes the WRC, which poises hypoxic cancer cells to respond to chemoattractants and increases their motility. While hypoxia is established as a driver of cancer metastasis, these data provide the first direct mechanism coupling hypoxia to the regulation of actin protrusions.

The biological and functional impact of PIM regulation of the WRC and plasticity of the actin cytoskeleton were profound. The negative impact of PIM inhibition on protrusive activity could be due to the relationship between ABI2 protein stability and WRC kinetics post-assembly. The WRC is assembled in the cytoplasm but remains inactive until bound by the Rho-family GTPase, Rac, at which point the VCA domain of WAVE is free to bind ARP2/3 and promote actin branching (Echarri et al., 2004; Mendoza, 2013). Once activated, the WRC translocates to the membrane, where it binds membrane receptors via a WRC interacting receptor sequence (WIRS) motif. The WIRS motif is composed of amino acids from both SRA and ABI2 and thus requires an intact WRC rather than individual subunits (Chen et al., 2013). Once bound to the membrane, ARP can then facilitate acting nucleation, branching, and ultimately the promotion of lamellipodia and protrusions (Leng et al., 2005; Rottner et al., 2021). Thus, it is possible that having more ABI2 present in the cell increases the binding of the WRC to the membrane via the WIRS motif. This is supported by the observation that localization of ARP3 at the leading edge was significantly impaired in cells lacking PIM, resulting in reduced protrusions, particularly in response to chemoattractants (Fig. 4, d and e). Future work is needed to determine how hypoxia and PIM1 impact the kinetics of WRC formation and disassembly, as the details of this process remain largely unknown.

Hypoxia increases metastasis through various mechanisms, including increased tumor angiogenesis, modification of extracellular matrix, and altered metabolism (Jensen and Warfel, 2021). However, little is known about the effect of hypoxia on cytoskeletal dynamics. Our work provides the first mechanistic evidence that hypoxia increases tumor cell invasion by enhancing WRC activation and cellular protrusion. Hypoxia has been shown to impact the expression and activity of nearly every major oncogene, both directly and indirectly, through HIF-1-driven changes in gene expression (Jensen and Warfel, 2021). As a result, efforts to target hypoxia therapeutically have focused on blocking HIF-1 activation, which has been largely unsuccessful. Therefore, targeting HIF-1-independent hypoxia-inducible signaling proteins, such as PIM1, presents a promising opportunity for therapy. Our findings indicate that the ability of hypoxia to enhance the invasive potential of PCa cells is largely dependent on PIM1. While PIM inhibitors are currently being tested in anticancer clinical trials, the benchmarks of these trials are focused on blocking tumor growth and inducing tumor cell death. This work provides compelling evidence for testing PIM inhibitors as antimetastatic agents in PCa. Further, the ability to distinguish between invasive and indolent tumors remains a long-standing clinical problem in PCa, as monitoring PSA levels is not a reliable indicator of tumor aggression (Mccaffery et al., 2019) and the field lacks well-defined subtypes. Our data suggest that PIM1 expression, in addition to ABI2, could serve as a potential biomarker to assess the invasive and metastatic potential of primary prostate tumors. Overall, this study provides exciting preclinical data that expands the potential utility of PIM inhibitors in PCa as antimetastatic agents that could reduce the invasive potential of prostate tumors, resulting in lower patient mortality.

## Materials and methods

### Cell lines and cell culture

Parental and genetically modified DU145 cells, HEK293T, WT, and TKO MEFs (Mikkers et al., 2004) cells were cultured in DMEM (Cat# 10-017-CV; Corning) containing 10% FBS (Omega Scientific). DU145 ABI2-knockout cell lines (hereafter referred to as ABI2-KO_A20_ and ABI2-KO_B76_) were generated by CRISPR-Cas9-mediated knockout using two CRISPR sgRNAs 5′-GCT​ACA​CTA​CAT​AAC​AAA​GG-3′ and 5′-TGT​GAT​TGA​GAT​TTA​CGA​TT-3′. Knockouts were confirmed by PCR, and positive clones were expanded for further use. PC3-LN4, PC3-LN4-pCIP, PC3-LN4-hPIM1, and PC3-LN4 PIM1-KO cells were cultured in RPMI 1640 medium (Cat# 10-040-CV; Corning) containing 10% FBS. MCF7 cells were cultured in high glucose DMEM base media with sodium pyruvate and l-glutamine (Cat# 10-013-CV; Corning) supplemented with an additional 100 mM l-glutamine (Cat# 25-005-CI; Corning), 10% FBS (Gibco), and antibiotics (100 U/ml penicillin + streptomycin; Cat# 30-002-CI; Life Technologies). All cells were cultured at 37°C in 5% CO_2_ with ambient O_2_ levels. Cells were routinely screened for mycoplasma and authenticated by the University of Arizona Genetics Core Facility and were used for <30 passages. Cells were cultured at 37°C in a hypoxic environment (1% O_2_, 5% CO_2_, 94% N_2_) using an InVivo2 400 hypoxia workstation (Baker Ruskinn) when stated. Lipofectamine 3,000 (Cat# L3000015; Thermo Fisher Scientific) was used to carry out transient transfection according to the manufacturer’s protocol. Immunoblotting and ubiquitination assays were performed as described previously (Warfel et al., 2011). ImageJ analysis software (National Institutes of Health) was used to perform densitometric analysis.

### Plasmids and siRNA

hPIM1 (short-form) was cloned into pCIP (lentiviral backbone) as previously described (Casillas et al., 2018). For the generation of ABI2 phospho-mutants, sequences containing the desired wild-type or mutant amino acids were created by TWIST biosciences and cloned into pGCS-N6 (Plasmid #85723; Addgene) using Gateway cloning. siRNA for ABI2 was purchased from Thermo Fisher Scientific (Cat# 4392420, siRNA ID s19769). GFP-WAVE was purchased from Addgene (Plasmid #54314). GFP-Lifeact and RFP-ARP3 plasmids were gifts from Dr. Ghassan Mouneimne (University of Arizona, Tucson, AZ, USA).

### Reagents and antibodies

Trypsin-EDTA (Cat# 25-053-CI), PBS (Cat# 21-031-CV), and laminin (Cat# 354232) were purchased from Corning. Recombinant EGF was purchased from Life Technologies (Cat# PHG0311). IGF was purchased from Sigma-Aldrich (Cat# I3769). PIM447 was purchased from Selleck Chemicals (Cat# S7985). AZD1208 was acquired from AdooQ Bioscience (Cat# A13203-750). DMSO was purchased from Thermo Fisher Scientific (Cat# 97064-724). Cycloheximide was purchased from VWR (Cat# 97064-724), and doxycycline was purchased from Sigma-Aldrich (Cat# D9891-5G). Recombinant myelin basic protein (MBP) was a gift from Dr. Greg Rogers, and recombinant ABI2 protein was purchased from Origene (Cat# TP300637). Radio-labeled ATP was purchased from Perkin Elmer (Cat# BLU502A). The antibody to ABI2 was purchased from Bethyl Laboratories (Cat# A302-499A-M). GFP (Cat# 2956S), HA (Cat# 3724S), HIF-1a (Cat# 14179S), p-IRS1 [S1101] (Cat# 2385S), PIM1 (Cat# 3247S), and WAVE2 (Cat# 3659S) antibodies were purchased from Cell Signaling Technology. The antibody for actin was purchased from BD Biosciences (Cat# 61656). PIM1 (Cat# ab75776) and Ki67 (Cat#ab833) antibodies used for immunohistochemistry were purchased from Abcam.

### Microscopy/image acquisition

Images were acquired using a Nikon Ti2 Inverted Microscope with Perfect Focus to correct for axial focal drift. A 60× oil objective with a .85 aperture from Nikon was used along with immersion oil from Nikon. The microscope is equipped with an environmental chamber that is heat-regulated and humidified and all images were taken at 37°C and 5% CO_2_ with varying concentrations of oxygen as noted in each experiment. All fluorescent images utilized fluorescently tagged proteins with GFP acquired using 488 nm laser excitation and ARP at 638 nm excitation using a SPECTRA X LED light engine. The camera used for image acquisition was CoolSNAP MYO and was run using the Nikon Elements Advanced Research Acquisition and Analysis Duo Package.

### Cell protrusion assays

Cells expressing GFP-Lifeact were starved (complete media with 0.5% FBS) for 4 h prior to imaging and allowed to equilibrate in the environmental chamber on the Nikon Ti2 microscope either at 20% or 1% O_2_ (37°C and 5% CO_2_ constant). Images were then taken every 15 s for 5 min at 60× magnification to establish a baseline. Imaging was paused to add 500 µl of media containing EGF or IGF to obtain the desired final concentration and then immediately resumed for an additional 10 min at 15-s intervals. The area of the cells was measured using Nikon Elements software and averaged for each cell during the baseline readings. The area of the cells after stimulation was also measured and plotted relative to that at baseline to determine the protrusive activity of the cell membrane.

### Image analysis

Unstimulated cells expressing GFP-Lifeact were starved (complete media with 0.5% FBS) and then imaged every 15 s for 15 min as described above. The resulting Nikon files were then uploaded into Matlab via the Bioformats package. Using code developed by Gaudenz Danuser (Lee et al., 2015), cell outlines were obtained, the edge of the cell was randomly divided into windows (10 μm wide), and protrusion vectors were generated based on the change in the cell outline between each frame. Each window of the cell was then sampled for the protrusion vectors that fell within it, and an average velocity per window was obtained. This was then displayed as a heat map. Further, the portion of the cell protruding at any given time could be determined based on the protrusion vector analysis, and a relative protrusive activity could be determined. We generated code to superimpose the outline of the cell at each given frame onto each other to form one image where each line represented the outline of the cell as a function of time.

### SILAC-based phosphoproteomics

SILAC was performed as described (Williams et al., 2016). Briefly, PC3-LN4 cells were grown subconfluently with isotopically distinct forms of arginine or lysine ([Arg0/Lys0], [Arg6/Lys4], or [Arg10/Lys8]) for approximately seven cell doublings. Once sufficient SILAC incorporation was confirmed by mass spectrometry, the cells were placed in normoxia or hypoxia for 8 h and then treated with AZD1208 (3 µM) or DMSO for 15 min. Biological replicates were performed with a label-swap control, and protein (5 mg) from each treatment group was combined at a 1:1:1 ratio and processed for LC-MS/MS. Two biological replicates were performed per condition. One sample *t* test was performed to compare the relative abundance of phosphopeptides between treatment conditions. To identify and quantify phosphopeptides, raw data were searched against a human Uniprot protein database downloaded on May 31, 2013, using MaxQuant v.1.5.1.0 (Max Planck Institute).

### Kinase assays and mass spectrometry

#### In vitro

Recombinant full-length GST-PIM1 was incubated with recombinant ABI2, GST, or MBP in the presence or absence of AZD1208 (100 nM) and ^32^P-labeled ATP in kinase assay buffer (20 mM MOPS [pH 7.0] containing 100 mM NaCl, 10 mmol/liter MgCl_2_, and 2 mmol/liter dithiothreitol). Reactions were incubated for 1 h at 37°C and subsequently separated by SDS-PAGE. Gels were stained with Coomassie and further analyzed via mass spectrometry or autoradiography.

#### In vivo

HA-ABI2 was immunoprecipitated with anti-HA antibodies. Immune complexes were washed with lysis buffer followed by kinase buffer and then incubated for 1 h with 100 ng of recombinant PIM1 in the presence or absence of AZD1208. Mass spectrometry was done following separation by SDS-PAGE and Coomassie staining. Sample preparation, LC-MS/MS parameters, and follow-up database searching were all performed as previously described (Casillas et al., 2021).

### Boyden chamber transwell assays

Transwell inserts (Cat# 353292; Corning) with a high-density PET membrane and 3-μm pore size were treated with PBS alone or PBS containing laminin (10 µg/ml) overnight to coat the wells. Wells were washed with PBS, and PC3-LN4 or DU145 cells in medium with 0.5% FBS were seeded directly into the Transwell inserts. Medium with 10% FBS was placed inside the lower chamber. The cells were allowed to invade overnight. The wells were then harvested, cells on the top side of the membrane were removed, and the bottoms of the inserts were stained using crystal violet (Cat# C581-25; Thermo Fisher Scientific). The number of invaded cells was measured to determine cellular migration (uncoated) and invasion (laminin-coated).

### 3D invasion assays

The 3D invasion assay was performed as previously described (Padilla-Rodriguez et al., 2018). Briefly, 1.0 × 10^5^ PC3-LN4 or PC3-LN4 with CRISPR knockout of PIM1 (PIM1-KO) cells were embedded in a central Matrigel matrix. The internal matrix was encapsulated by an external collagen matrix, and the cells were allowed to migrate into the outer collagen matrix for 48 h. The cells were then fixed with 4% paraformaldehyde and stained with Hoechst 33342 for 24 h at 4°C. Confocal z-series of Hoechst and differential interference contrast images were acquired at 1.5-μm z-steps on a Nikon Ti-E inverted microscope. A large stich composite image of the entire area, including the internal dot and invaded cells, was generated. The number of invaded cells was quantitated using Nikon Elements as was the invasion distance for each nucleus.

### Smooth muscle invasion assay

All animal studies were approved by the Institutional Animal Care and Use Committee of the University of Arizona. Approximately one million prostate tumor cells (DU145 or ABI2-KO_A20_) in sterile saline were injected into the peritoneal cavity of 6–8-wk male SCID mice. After 2 wk, mice were randomly segregated into groups for treatment with vehicle or the PIM inhibitor PIM447 (30 mg/kg, p.o. daily). 4 wk after injection, mice were euthanized by cervical dislocation to avoid changes in tissue oxygenation due to artifacts of euthanasia by CO_2_. After euthanasia, the diaphragm from each mouse was collected, fixed in formalin, and processed for histology. Stained slides were digitally scanned using an Aperio Image Scanner. Scanned slides were subsequently analyzed to obtain the depth of invasion (distance nuclei traveled into the diaphragm), tumor burden (a portion of the diaphragm with tumor present), and tumor penetrance (a portion of the seeded tumor that exhibits invasion into the diaphragm).

### ARP kymography assay

WT and TKO MEF cells with stably expressed GFP-Lifeact and RFP-ARP3 were serum-starved and placed in hypoxia for 4 h prior to the addition of PDGF for 30 min. Kymographs were generated by starting at the cell edge and going proximal to the center of the cell parallel to the actin filaments. The intensity of ARP3 staining was plotted relative to the distance from the cell edge.

### Immunoprecipitation assays

For coimmunoprecipitation assays, DU145 or PC3-LN4 cells were transfected with the indicated constructs. Then, cells were treated as stated and placed in hypoxia for the stated times. Cells were harvested in an IP lysis buffer (20 mM Tris HCl, pH 8; 137 mM NaCl; 10% glycerol; 1% Nonidet P-40; and 2 mM EDTA) with protease inhibitors and centrifuged at 15,000 RPM for 10 min. Lysates were incubated overnight at 4°C with HA magnetic beads (Cat# 88836; Pierce) or GFP magnetic beads (Chromotek) and analyzed by Western blotting as described above.

### Statistical analysis

A minimum number of three replicates were performed to ensure adequate statistical power for all in vitro experiments. One-way ANOVA and unpaired *t* test were performed, and P < 0.05 was considered statistically significant. Confidence intervals are 0.95 (α = 0.05). Figure legends contain all information regarding “*n*” values and P values.

### Online supplemental material

Fig. S1 contains data pertaining to the effect of chemical and genetic loss of PIM on cell spreading and migration. Fig. S2 demonstrates the direct phosphorylation of ABI2 by PIM1 in vitro and analysis of gene expression levels of ABI1 during PCa progression. Fig. S3 contains data showing the requirement of ABI2 for PIM1 to increase WAVE2 in a second CRISPR-ABI knockout cell line. Fig. S4 contains data related to the effect of PIM and ABI2 loss on cell migration and wound healing. Fig. S5 shows IHC staining for cell proliferation from different cell line models used for in vivo diaphragm invasion assays.

## Supplementary Material

SourceData F3 is the source file for Fig. 3.

SourceData F4 is the source file for Fig. 4.

SourceData FS3 is the source file for Fig. S3.

SourceData FS4 is the source file for Fig. S4.
