# Supplementary figures and images for: PIM1 phosphorylates ABI2 to enhance actin dynamics and promote tumor invasion
Source: J Cell Biol. 2023 Apr 12;222(6):e202208136. doi: 10.1083/jcb.202208136 (PMC10103708; doi:10.1083/jcb.202208136)

Fig 3 A

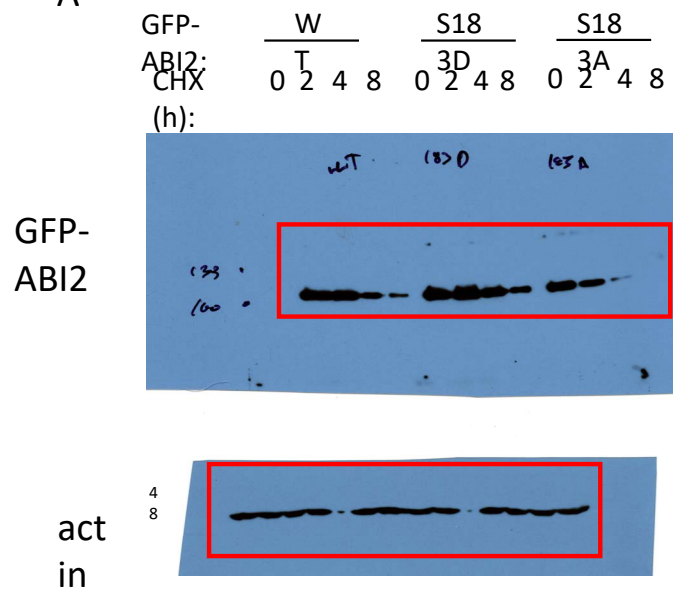

B

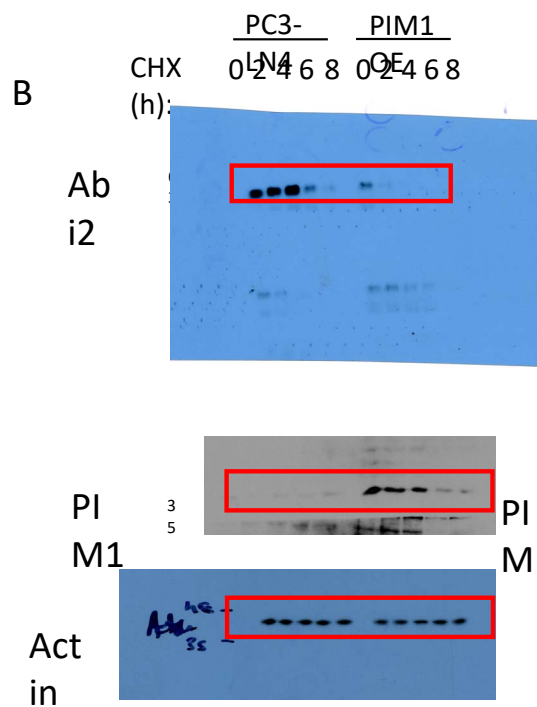

C

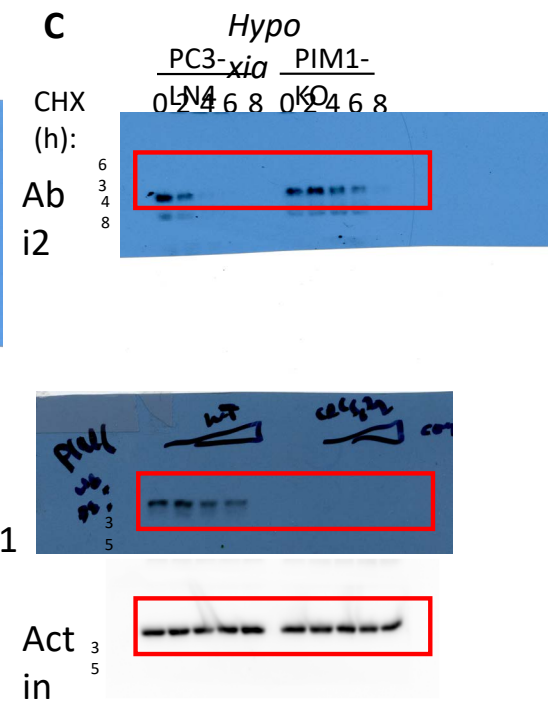

Supplement: SourceData F3 — is the source file for Fig. 3. [file JCB_202208136_SourceDataF3.pdf]

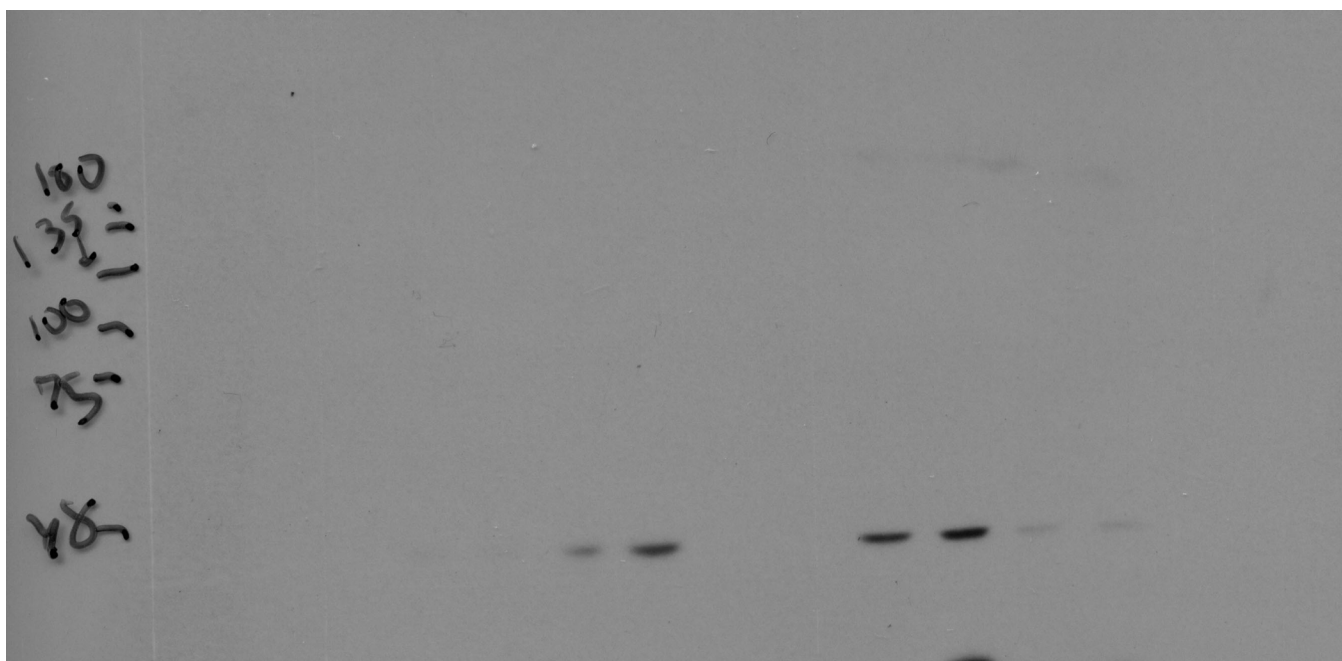

Supplement: SourceData F4 — is the source file for Fig. 4. [file JCB_202208136_SourceDataF4.pdf]

|       | DU145 |   | B76 |   |
|-------|-------|---|-----|---|
| PIM1: | -     | + | -   | + |

ABI2

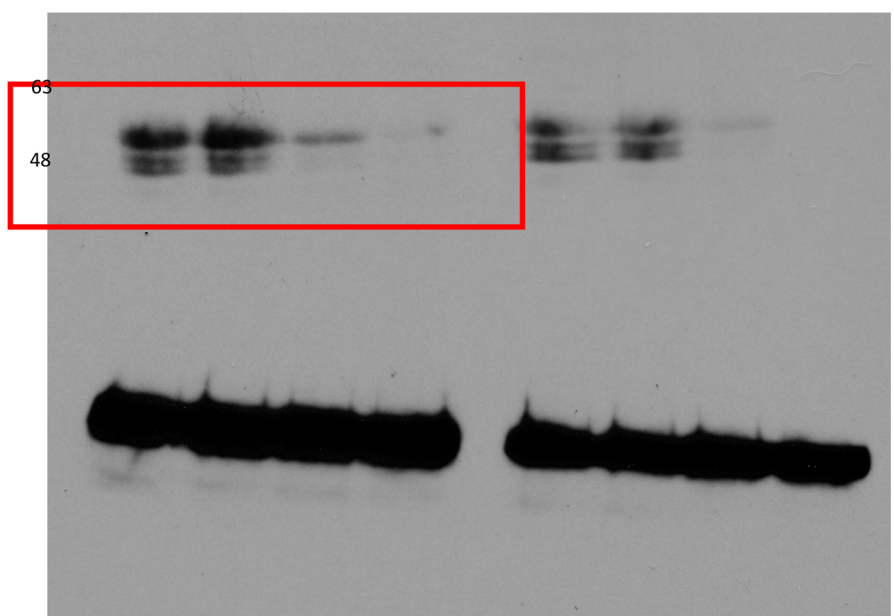

Fig S3

PIM1

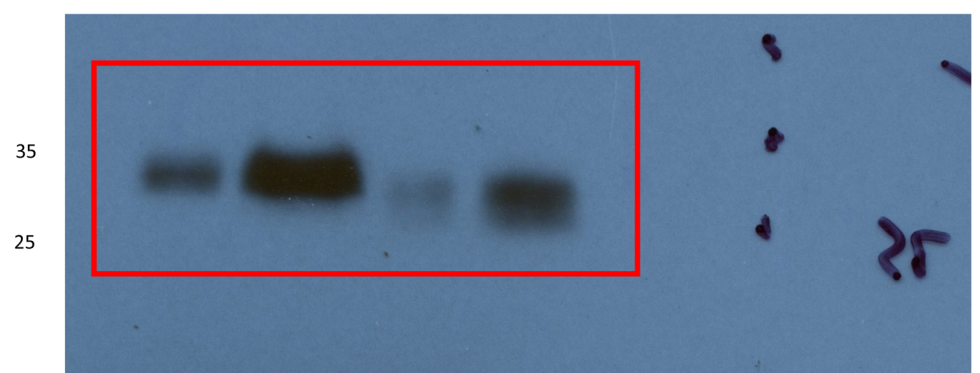

WAVE2

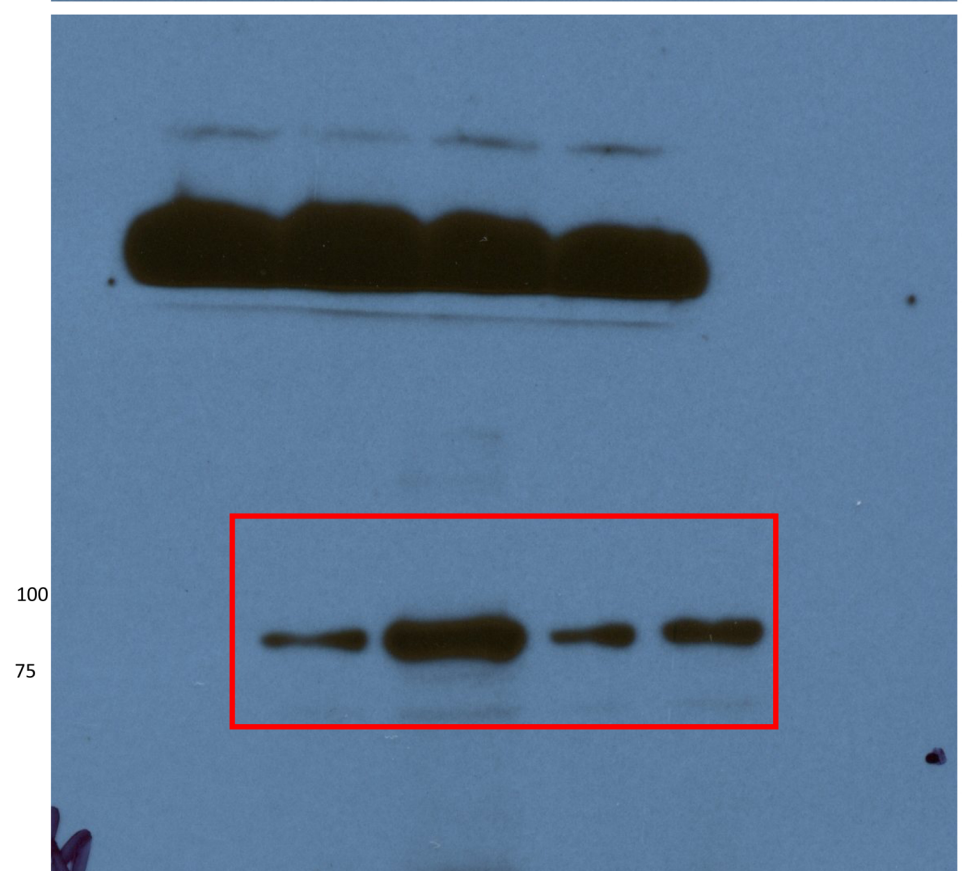

ACTIN

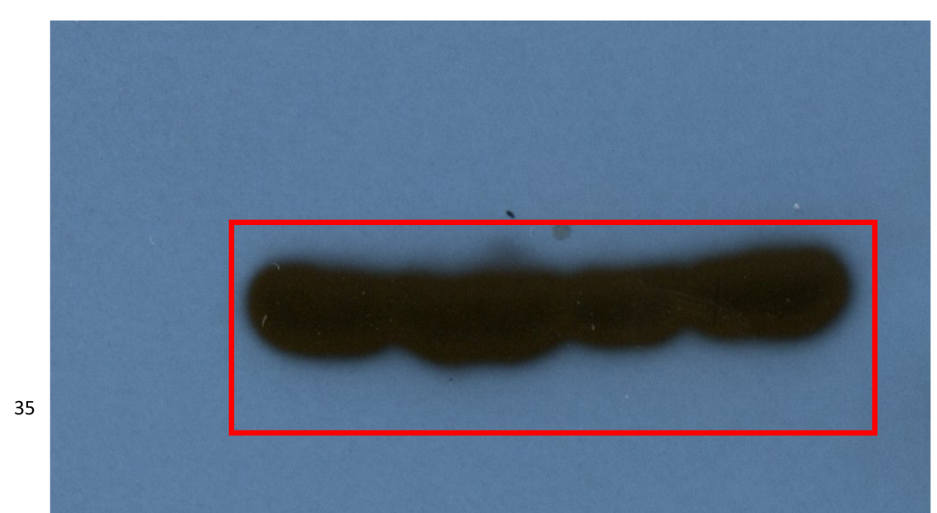

Supplement: SourceData FS3 — is the source file for Fig. S3. [file JCB_202208136_SourceDataFS3.pdf]

Fig S4

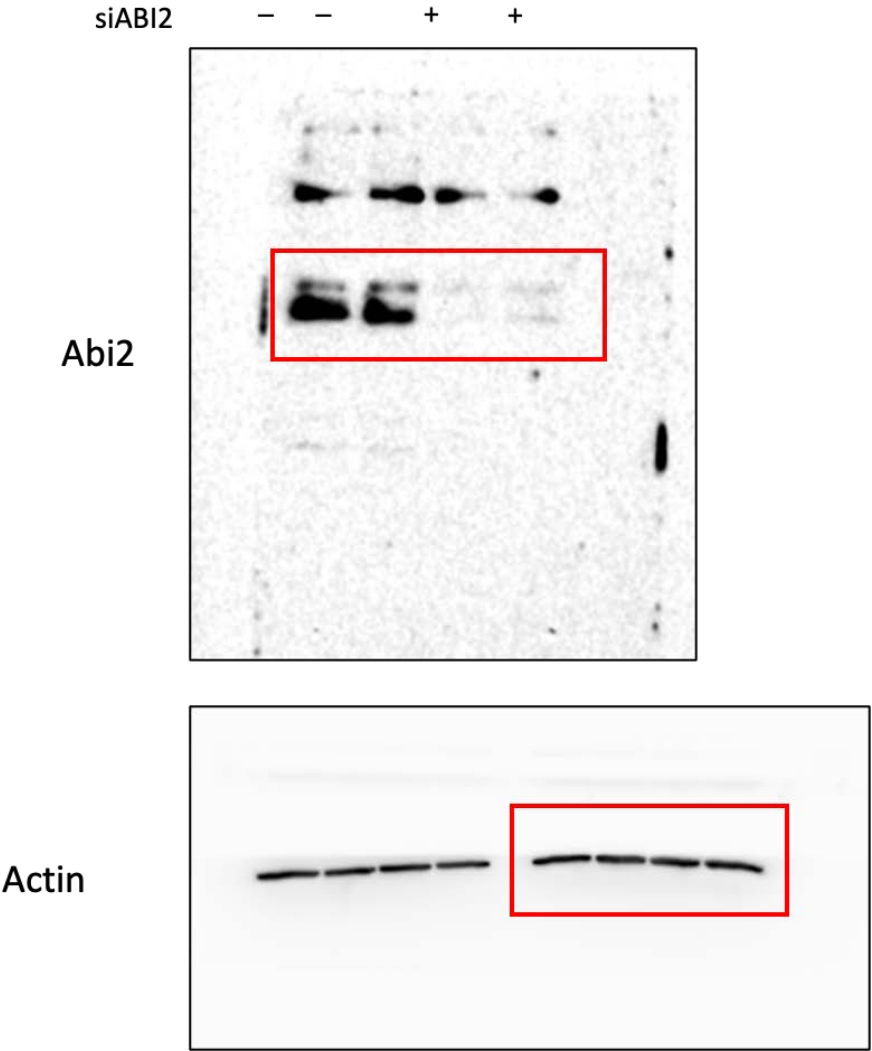

Supplement: SourceData FS4 — is the source file for Fig. S4. [file JCB_202208136_SourceDataFS4.pdf]
